# Pathologic gene network rewiring implicates PPP1R3A as a central regulator in pressure overload heart failure

**DOI:** 10.1038/s41467-019-10591-5

**Published:** 2019-06-24

**Authors:** Pablo Cordero, Victoria N. Parikh, Elizabeth T. Chin, Ayca Erbilgin, Michael J. Gloudemans, Ching Shang, Yong Huang, Alex C. Chang, Kevin S. Smith, Frederick Dewey, Kathia Zaleta, Michael Morley, Jeff Brandimarto, Nicole Glazer, Daryl Waggott, Aleksandra Pavlovic, Mingming Zhao, Christine S. Moravec, W. H. Wilson Tang, Jamie Skreen, Christine Malloy, Sridhar Hannenhalli, Hongzhe Li, Scott Ritter, Mingyao Li, Daniel Bernstein, Andrew Connolly, Hakon Hakonarson, Aldons J. Lusis, Kenneth B. Margulies, Anna A. Depaoli-Roach, Stephen B. Montgomery, Matthew T. Wheeler, Thomas Cappola, Euan A. Ashley

**Affiliations:** 1 Division of Cardiovascular Medicine, Stanford University, Stanford, CA 94305 USA; 2 Biomedical Informatics Program, Stanford University, Stanford, CA 94305 USA; 3 Department of Pathology, Stanford University, Stanford, CA 94305 USA; 4 Division of Cardiology, University of Pennsylvania, Philadelphia, PA 19104 USA; 5 Department of Medicine, University of Pennsylvania, Philadelphia, PA 19104 USA; 6 Department of Medicine, Boston University School of Medicine, Boston, MA 02118 USA; 7 Department of Pediatrics, Stanford University, Stanford, CA 94305 USA; 8 Department of Cardiovascular and Metabolic Sciences, Lerner Research Institute, Cleveland, OH 44195 USA; 9 Department of Cardiovascular Medicine, Heart and Vascular Institute, Cleveland, OH 44195 USA; 10 Providence Medical Group—Milwaukie, Portland, OR 97222 USA; 11 Center for Bioinformatics and Computational Biology, University of Maryland, College Park, MD 20740 USA; 12 Biostatistics and Epidemiology, University of Pennsylvania, Philadelphia, PA 19104 USA; 13 Department of Pathology, University of California San Francisco, San Francisco, CA 94143 USA; 14 Department of Pediatrics, The Children’s Hospital of Philadelphia, Philadelphia, PA 19104 USA; 15 Departments of Medicine, University of California Los Angeles, Los Angeles, CA 90095 USA; 16 Perelman School of Medicine, University of Pennsylvania, Philadelphia, PA 19104 USA; 17 School of Medicine, Indiana University, Indianapolis, IN 46202 USA; 18 Department of Genetics, Stanford University, Stanford, CA 94305 USA; 19 Center for Undiagnosed Diseases, Stanford University, Stanford, CA 94305 USA

**Keywords:** Cellular signalling networks, Gene expression, Heart failure, Cardiovascular genetics

## Abstract

Heart failure is a leading cause of mortality, yet our understanding of the genetic interactions underlying this disease remains incomplete. Here, we harvest 1352 healthy and failing human hearts directly from transplant center operating rooms, and obtain genome-wide genotyping and gene expression measurements for a subset of 313. We build failing and non-failing cardiac regulatory gene networks, revealing important regulators and cardiac expression quantitative trait loci (eQTLs). *PPP1R3A* emerges as a regulator whose network connectivity changes significantly between health and disease. RNA sequencing after *PPP1R3A* knockdown validates network-based predictions, and highlights metabolic pathway regulation associated with increased cardiomyocyte size and perturbed respiratory metabolism. Mice lacking *PPP1R3A* are protected against pressure-overload heart failure. We present a global gene interaction map of the human heart failure transition, identify previously unreported cardiac eQTLs, and demonstrate the discovery potential of disease-specific networks through the description of *PPP1R3A* as a central regulator in heart failure.

## Abstract (web summary)

The genetic and pathogenetic basis of heart failure is incompletely understood. Here, the authors present a high-fidelity tissue collection from rapidly preserved failing and non-failing control hearts which are used for eQTL mapping and network analysis, resulting in the prioritization of *PPP1R3A* as a heart failure gene.

## Introduction

Heart failure (HF) is a life-threatening syndrome characterized by an inability of the heart to meet the metabolic demands of the body. HF costs the US more than $34 billion a year to treat 6 million patients^1,2^. Despite this, the underlying molecular mechanisms remain poorly understood and the few approved therapeutics target maladaptive compensatory pathology rather than proximate molecular mechanisms^3,4^.

With rapidly increasing access to high-throughput sequencing technology, molecular characterization of human heart tissue has become possible, and in recent years a number of efforts to define the regulatory transcriptional architecture of HF in humans and small animals have been undertaken^5–12^. These efforts have revealed changes in gene expression of key sarcomeric, calcium cycling, developmental and metabolic genes. Because of the significant logistical challenge of harvesting healthy hearts, few studies have included a nonfailing control group, making conclusions regarding the transition to HF tentative. Further, as gene expression programs are rapidly altered in an environment of high oxidative and nitrosative stress^13^, the high metabolic rate of the heart limits the utility of post-mortem tissue for gene expression analysis (e.g., from public resources such as GTEx^14–16^).

Thus, the expansion of this resource with high-fidelity tissue collection and molecular characterization is required, and a crucial next step in synthesizing this information is the identification, and in vitro and in vivo validation, of novel molecular actors in this disease. In this study, we identify previously undetected cardiac expression quantitative trait loci (eQTLs) from genome-wide genotyping and gene expression measurements from rapidly preserved failing and nonfailing human heart tissue. Condition-specific cardiac regulatory gene networks identify disease-driven changes in local and global topology and pathway organization, illuminating *PPP1R3A* as a novel predicted HF regulator. Lastly, our in vitro and in vivo approaches further demonstrate its role in HF pathology.

## Results

### Immediate tissue processing yields quality transcriptomic data

**Fig. 1:**
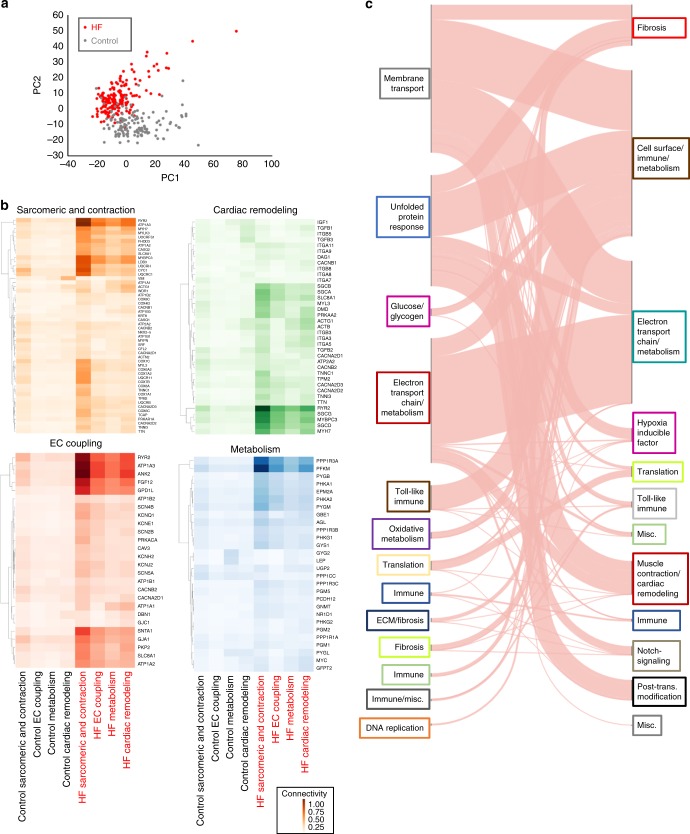
Regulatory rewiring of coexpression networks in HF. **a** Principal component analysis of gene expression profiles for 177 failing hearts and 136 nonfailing, control, hearts showing clear segregation of HF (red) vs. control (gray) population. **b** Differential connectivity of known biological processes in HF. Normalized connectivity (sum of WGCNA weights divided by maximum network weight) between representative genes from four known processes that play critical roles in HF (sarcomeric and contraction genes (orange), EC coupling (red), cardiac remodeling (green), and metabolism (blue)) to all genes from those same processes in HF and controls. Genes of each process are rows and columns are process and cohort. For example, *MYBPC3* in the cardiac remodeling process (third row in green heat map) is highly connected to sarcomeric and contraction genes and cardiac remodeling genes in the HF network (fifth and eighth columns respectively) compared to all the control processes. **c** River plot demonstrating changing modular assignments for genes in the HF vs. control networks. Pink lines represent individual genes, with left-sided grouping representing membership in control (left) and HF (right) network modules (indicated by color of text box) and right-sided grouping in HF network modules, with text indicating module names derived from KEGG and Reactome associations of genes within each module

The MAGnet consortium was founded to establish best practices for the harvesting of human cardiac tissue (see Methods) and to explore the genetic landscape of cardiac gene expression^7,13,17^. Using this consensus protocol, we obtained 1352 human cardiac samples and chose a subset of 313 hearts, including 177 failing hearts collected immediately post transplantation and 136 healthy donor controls that were suitable for transplantation but did not reach a recipient due to logistical reasons (clinical characteristics listed in Supplementary Table 1). We genotyped and measured left-ventricular genome-wide gene expression of these samples, controlling for known covariates, specifically age, gender and collection site. Principal component analysis showed that additional covariates do not explain a significant proportion of variance in gene expression (Fig. 1a and Supplementary Fig. 1).

We assessed the quality of these measurements in several ways. First, we found that disease status was the dominant source of variation suggesting no major confounding sources of variation (Supplementary Fig. 1). Second, we confirmed enhanced expression of *NPPA* and *NPPB*, depletion of *SERCA2A*, and a shift from *MYH7* towards *MYH6* expression—established signatures of HF (Supplementary Fig. 1A and B). In total, 793 genes were significantly upregulated in failing hearts compared to nonfailing and 848 were downregulated (fold change greater or lesser than 2 or 0.5 respectively, with FDR < 0.01; full differential expression analysis is available in Supplementary Data 3 and 4). Finally, as our sample collection was performed immediately before or after cardiac transplantation (unlike post-mortem samples such as those used in GTex), we investigated whether gene expression programs related to oxidative stress were less perturbed than in samples collected post mortem. To do this, we compared oxidative stress gene expression as defined by genes in the GO term “Response to oxidative stress”(GO:0006979) in our samples to left-ventricular sample data in GTEx, as well as other KEGG and Reactome pathways (data obtained from the recount2 database^18^). Our results suggested that our samples displayed comparable contractility-related gene expression but had significantly less oxidative-stress-related gene expression and less perturbation in other metabolic pathways (Supplementary Fig. 2C). Having established the quality of our data, we limited our network-based downstream analyses to the 40% genes most variably expressed between failing and nonfailing hearts (*n* = 7960) in order to limit inflation of correlation between low covariance gene pairs.

### Cardiac coexpression maps reveal dynamic network topology

We inferred undirected, disease-state-specific gene coexpression networks. Gene regulatory network inference from coexpression is a challenging problem that no single method solves adequately in all contexts. Here, we constructed control- and HF-specific networks using methods that rely on gene coexpression (Weighted Gene Co-expression Network Analysis (WGCNA)^19,20^ and Pearson correlation), inverse covariance estimation (Joint Graphical LASSO (JGL)^21^), and mutual information (ARACNe^22^ and ZScore) (see Methods for details. All network constructions are available at 10.5281/zenodo.2600420). Each of these methods has specific advantages for different questions, e.g. JGL creates a sparser network with the specific intent of reducing representation of noncausal associations, whereas WGCNA relies on denser network topology to capture modules of genes with high likelihood of interaction. Since our downstream analysis required a top-down systems view of coexpression networks, and because we planned to prioritize genes based on the change in connectivity of networks between disease states, we chose to base our subsequent analyses on WGCNA-derived networks, which represent a robust tool for this purpose.^23,24^

To achieve an initial understanding of topological changes between the nonfailing and failing heart networks, we compared the structure of modules in each WGCNA-derived network (dendrograms used for module finding shown in Supplementary Fig. 3). These networks displayed different structure in HF than in control: First, the number of genes unassigned to any module was much fewer in HF (13 genes were unassigned, compared to 2614 in controls). Second, while each group of genes was specifically enriched with functional annotations as revealed by enrichR^25^, the HF modules had more diversity of signaling and metabolic annotations (full gene module descriptions and Benjamini−Hochberg-adjusted enrichment *p* values are shown in Supplementary Data 5 and 6).

We then manually curated modules of genes related to four key processes involved in HF (Fig. 1b: sarcomeric and contraction genes (orange), excitation−contraction (EC) coupling (red), cardiac remodeling (green), and metabolism (blue), see Supplementary Data 11). Network connectivity changed within these process-based modules between nonfailing and failing networks. Compared to the nonfailing network (gray typeface), the failing heart network (red typeface) saw a general rewiring in connectivity within and between these modules; metabolic genes gained a few specific genes such as the protein phosphatase 1 catalytic and regulatory subunits (*PPP1CC, PPP1R1A*, and *PPP1R3A/B/C*) and the muscle 6-phosphofructokinase *PFKM* in the HF network (Fig. 1b, blue). Cardiac remodeling genes that gained connectivity were *MYBPC3*, *MYH7*, *RYR2*, and *SGCG/D*; sarcomeric and contraction genes that gained connectivity were *MYBPC3, MYH7*, again listed due to pathway overlaps and *VIM, UQCRH/C1*; while for EC coupling these were *ATP1A1/2/3* and again *RYR2*.

Finally, plotting a Sankey diagram to observe where module membership changes from controls to HF (Fig. 1c) revealed large rewiring of coexpression structure. Shared core structure modules such as electron transport chain (ETC) and metabolism genes mostly remained in the same module (dark red in controls and turquoise in HF), while the unassigned genes in the controls (gray) went mostly to the metabolism/ETC (turquoise), cell surface/immune/metabolism (brown), and fibrosis (red) modules in HF.

### High-quality tissue expression data reveal cardiac eQTLs

**Fig. 2:**
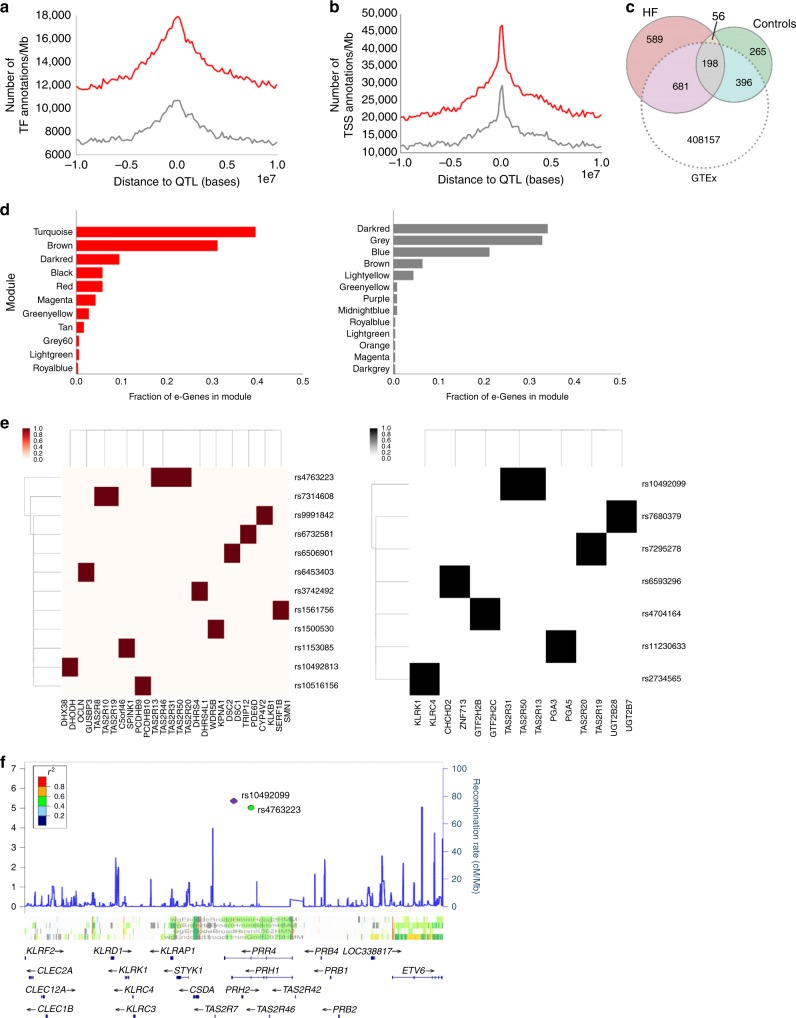
High-quality tissue expression reveals previously undetected cardiac eQTL associations. Transcription factor (**a**) and transcription start site (**b**) annotation to eQTL distance distributions for failing (red) and control hearts. **c** Number of *cis* eQTLs found for each group that overlapped with GTEx eQTLs. **d** Fraction of modules with genes found to be controlled by at least one eQTL in HF (red, left) and controls (gray, right). **e** Heat map indicators for variants controlling multiple genes *in-cis* in HF (red, left) and controls (gray, right). In the rows are SNPs controlling genes (columns) colored intensely if the SNP controls the gene. **f** Variants from one locus control a network of G-protein-coupled receptors *TAS2R* present in both the failing and control groups

We then leveraged genome-wide genotypes to find gene-expression-controlling loci (eQTLs) in each cohort. First, we performed hidden covariate correction using the PEER package (see Methods). We used QTLtools to perform association testing for each cohort separately (see Methods) and performed a scan on the number of hidden factors to correct with PEER.^26^ We found that the HF cohort had more associated eQTLs than the control group (1566 vs. 936, respectively, with an overlap of 254 loci between the two groups, see 10.5281/zenodo.2617028 for full eQTL results); as expected these eQTLs showed proximity to known transcription factor binding sites and transcription start sites (Fig. 2a, b). We then tested these eQTLs for enrichment of regulatory associations using RegulomeDB, a database of known and predicted regulatory regions of the genome^27^. Here, both cohorts had several eQTLs with adjacent (within 50 base pair window) regulatory annotation (716/1566 (46%) and 425/936 (45%) of variants, for failing vs. control, respectively) and/or predicted for transcription factor binding (Supplementary Fig. 4A). We then compared our eQTLs with those found by the GTEx project. Our set of eQTLs contained hundreds of novel associations when compared to the GTEx database for left-ventricular tissue: 831/1566 (53% novel associations) for the HF group and 423/936 (45% novel associations) for the control group (Fig. 2c). We also identified significant overlap with specific tissue types (e.g. artery vs. muscle) and cell types (e.g. cultured fibroblasts) (Supplementary Fig. 4B). Comparison to a recently updated version of GTEx yielded even greater overlap (Supplementary Table 2).

This percentage of newly identified eQTLs is larger than on previous, related eQTL studies^10,28^, and also displays greater overlap with GTEx than those found in an independent cohort of patients with dilated cardiomyopathy^10^. This large overlap was encouraging, and since we control for known and hidden covariates, our difference in eQTLs compared to related studies may reflect the immediate tissue collection techniques we used, and the difference in disease status (at least one third of cases in this study had ischemic disease, which was not true for comparison studies, which focused on nonischemic dilated cardiomyopathy^10^).

To assess the physiological impact of our eQTLs, we checked for overlap of our eQTL associations with existing variants in the GWAS catalog^29^. First, we did a simple, direct SNP overlap check with the GWAS variants (GWAS catalog variants with an LD cutoff of 0.6, using SNiPA^30^ to check for LD overlaps). For HF eQTLs, this revealed 41 variants, among which we found 25 associations with sudden cardiac arrest, heart rate variability, and coronary heart disease among other diseases/traits, whereas 33 of the nonfailing control eQTLs had associations in the catalog, including QT interval and heart rate variability traits (Supplementary Data 7 and 8). To assess whether the magnitude of this overlap was higher than expected, we used SNPSNAP^31^ to generate two sets of 10,000 random variants each with the same LD and gene density characteristics as our failing and nonfailing eQTLs. The average overlap of these random sets to GWAS variants was 0.1 for both sets, yielding empirical *p* values of less than 0.001 in each set and confirming that our overlap is higher than expected.

To expand our analysis of overlap of our eQTL findings with GWAS, we used eCAVIAR^32^, a high-resolution method that leverages SNP density to perform colocalization enrichment tests between hits in a high-powered, publicly available coronary artery disease GWAS and nearby eQTLs from our analysis (see Methods). We chose this coronary artery disease GWAS for comparison not only because it is one of few related to causes of HF that are appropriately powered for this high-resolution method, but believed it to be a reasonable disease surrogate for comparison based on the 36% of explanted failing hearts in our study that had undergone coronary artery bypass grafting (Supplementary Table 1). This method found seven regions nearby seven genes that have coronary artery disease-associated SNPs and that are significantly colocalized with our eQTLs (see Methods).

We then interrogated which gene modules uncovered by WGCNA were controlled by eQTL loci in concert by examining the fraction of genes that were e-genes of in the eQTL analysis (Fig. 2c). The modules with the top fraction of e-genes was turquoise in HF and dark red in the controls (40% and 32%, respectively), both of which correspond to ETC and metabolism genes. In HF, the next modules with the most fraction of e-genes were the brown module, a combination of cell surface, immune, and metabolism genes, as well as the dark red module, comprised of muscle contraction and cardiac remodeling genes. For the control cohort, the next module most enriched with e-genes was the gray/unassigned module, indicating a less cohesive regulatory structure; then followed by the blue module dominated by unfolded protein response genes.

We went on to identify modules of coordinating genetic loci and associated networks of genes within these associations by finding nontrivial connected components (i.e. with more than three nodes) within the bipartite association graph of variants and genes, including WGCNA edges with weights larger than the median (Fig. 2d). Notably, we found two eQTLs within a region enriched with predicted histone modifications that controlled a network of several *TAS2R* members *in-cis*, a family of G-protein-coupled receptors, in both failing and control groups (Fig. 2e, f, r10492099 and rs4763223). As *TAS2R* receptors can have high homology in some regions, we checked for potential probe cross-hybridization. We compared sequences for all *TAS2R* gene probes for the GeneChip ST1.1 array by BLASTing them against human transcript sequences (evalue cutoff of 0.01, with at least 12/25 exact matches). The genes *TAS2R43*−*TAS2R46* had overlapping, high similarity matching probes, suggesting possible cross-hybridization, while the rest of the receptors’ probes were deemed independent by this analysis. These associations, prevalent in both cohorts, highlight a common module of G-protein-coupled receptors that have been previously observed to be expressed in the healthy and failing heart^33,34^, and that may play a role in the regulation of arrhythmia and contractility^35^.

In summary, our heart transplant cardiac samples and inferred gene coexpression networks enabled us to find several previously unidentified cardiac eQTLs in the failing and nonfailing heart. Many of the eQTL variants were also associated with cardiac phenotypes in GWAS and some are associated with genes in highly connected parts of the coexpression network, suggesting coordinated regulation.

### Dynamic network topology illuminates central HF regulators

Next, we used our network topology to identify and prioritize genes that were dynamically connected between the failing and control heart networks. Our goal was to identify genes whose network connectivity was increased in the disease state (i.e. HF), but specifically to pathways known to be relevant to the global control of HF mechanisms. We achieved this by ranking genes on two connectivity metrics: (i) differential local network connectivity for each gene between control and failing networks, and (ii) change in each gene’s connectivity globally to HF-relevant molecular pathways manually curated from KEGG and Reactome (curated list of pathways included in Supplementary Data 9). We defined local connectivity (LC) as the change in number of edges for each gene between the control and HF networks. Global connectivity (GC) was defined as the number of curated HF-relevant pathways to which each gene was significantly differentially connected in the control vs. HF networks, taking into account network distance (see Methods, Supplementary Fig. 5).

**Fig. 3:**
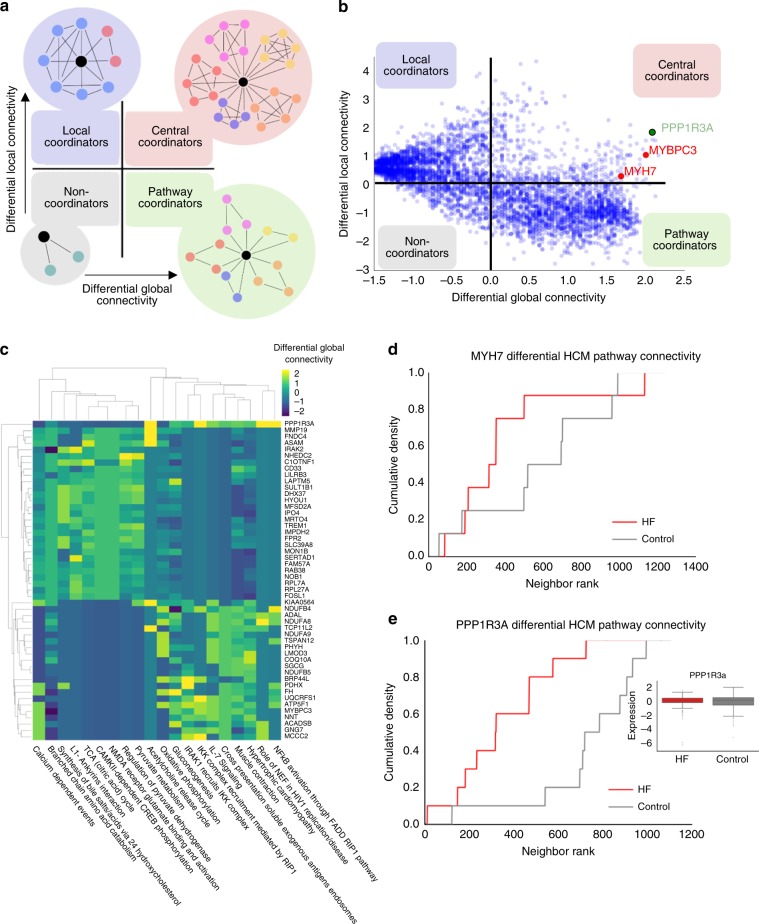
Gene prioritization through network topology. **a** Diagrammatic representation of roles of local and global connectivity in defining each gene’s coordinator status. Local connectivity (LC) is the per-gene change in coexpression edges in the HF vs. control network. Global connectivity (GC) represents the enrichment of HF-relevant pathways in a gene’s neighborhood between HF and control networks (see Methods). **b** Change in local and global connectivity for all genes between control and HF networks identified *PPP1R3A* (green font) as a central coordinator in HF, indicating its increased association with HF-relevant pathways as well as coexpression relationships in the HF vs. control networks. **c** Gene-pathway (rows-columns) differential connectivity matrix for genes ranked highest for global network connectivity that is differentially increased between non-heart-failure and heart-failure conditions. Differential connectivity is measured by the KS statistic between the distribution of ranks for pathway genes in HF and non-heart-failure conditions. In particular, metabolism, HF and cardiomyopathy pathways (yellow indicates an increase in connectivity to the given pathway (columns) in HF compared to control samples). **d** Difference in cumulative membership distributions for the KEGG Hypertrophic Cardiomyopathy (HCM) pathway for the myosin gene *MYH7* which is known to be involved in HCM. **e** Difference for HCM pathway enrichment in the protein phosphatase 1 regulatory subunit *PPP1R3A* is more dramatic than for *MYH7*. Inset: *PPP1R3A* transcriptional expression in HF and Control cohorts is unchanged

Using GC and LC, we assigned each gene to one of four categories (Fig. 3a): Noncoordinators were genes with decreased LC and GC between the control and HF networks, making them less likely to be highly impactful in the disease state. Local coordinators had significant increases in LC but decreased GC, indicating high coexpression, but mostly with genes unrelated to global HF processes. Pathway coordinators were genes with increased GC but overall low LC, indicating an increased association with global HF processes, but overall low impact with respect to gains in coexpression. Finally, central coordinators increased both in GC and LC, and therefore represent genes with increased local coexpression involving an increased number of global HF-relevant processes (a list of genes and assigned status are included in Supplementary Data 10).

After classifying each gene in this way, we focused on central coordinators (Fig. 3b, top right) in order to capture those genes with the most dynamic connectivity in disease. In addition to increased connectivity with HF-relevant pathways in the HF vs. control networks, these central coordinators were enriched in OMIM/KEGG cardiomyopathy terms and pathways (hypertrophic and dilated cardiomyopathy KEGG pathway and OMIM terms, Fisher exact test *p* values < 0.001). This includes the myosin binding protein C3 (*MYBPC3*) which has previously been implicated in the Mendelian cardiac muscle diseases, hypertrophic cardiomyopathy and dilated cardiomyopathy^36,37^ (Fig. 3b). Particularly, we noted that genes with highest increases in global connectivity regulated HF-related pathways across several cardiomyocyte-relevant processes including metabolism, muscle contraction, and cardiomyopathy-related genes (Fig. 3c). These data are in concordance with transcriptomic data from murine myocardium with and without exposure to transaortic constriction, which revealed prevalent gene modules associated with mitochondrial and cytoskeletal gene ontologies.^28^ In contrast, prioritization by differential gene expression between failing and control myocardium did not reveal many genes genetically associated with known cardiovascular disease pathways. We found no relationship between differential expression and either global or local differential connectivity. Only 1 of the top 20 highly differentially expressed genes was associated with cardiovascular disease pathways in KEGG or Reactome compared to 5 out of the top 20 for the connectivity-derived list—a significant enrichment difference ((Fisher exact test *p* value < 0.001), Supplementary Fig. 2).

Among those central coordinators whose network connectivity was maximally changed in the failing heart was protein phosphatase 1 regulatory subunit 3A (*PPP1R3A*), with one of the highest changes in GC between control and failing hearts (green typeface, Fig. 3b). *PPP1R3A*, which encodes a muscle-specific regulatory subunit of protein phosphatase 1 (PP1)^38^, has not been previously associated with HF. To examine the importance of *PPP1R3A* to cardiomyocyte hypertrophy across cardiomyopathic etiologies, we also examined its importance in a cardiomyopathy pathway (hypertrophic cardiomyopathy, KEGG), and found that its differential connectivity to this pathway (Fig. 3d) exceeded even that of *MYH7* (Fig. 3e), an exemplar cardiomyopathy gene. Additionally, we noted that connectivity of *PPP1R3A* to our lists of sarcomeric and contraction genes was increased significantly in HF (Fig. 2b). Previous work indicates *PPP1R3A* contains a glycogen-binding domain^39^ and is thought to promote skeletal muscle glycogen synthesis, and variants in *PPP1R3A* have been associated with decreased insulin sensitivity^40,41^. Our own networks showed increased connectivity of *PPP1R3A* to metabolic pathways in control and failing myocardium as well (Figs. 1b, 3c). As cardiac metabolism in HF is known to switch toward a glucose-based metabolism, and as metabolic pathways were significantly connected to *PPP1R3A* in our HF network (Fig. 3c), we hypothesized that this gene would play an important role in the transition from healthy to failing myocardium.

### PPP1R3A knockdown ablates HF phenotypes in vitro

**Fig. 4:**
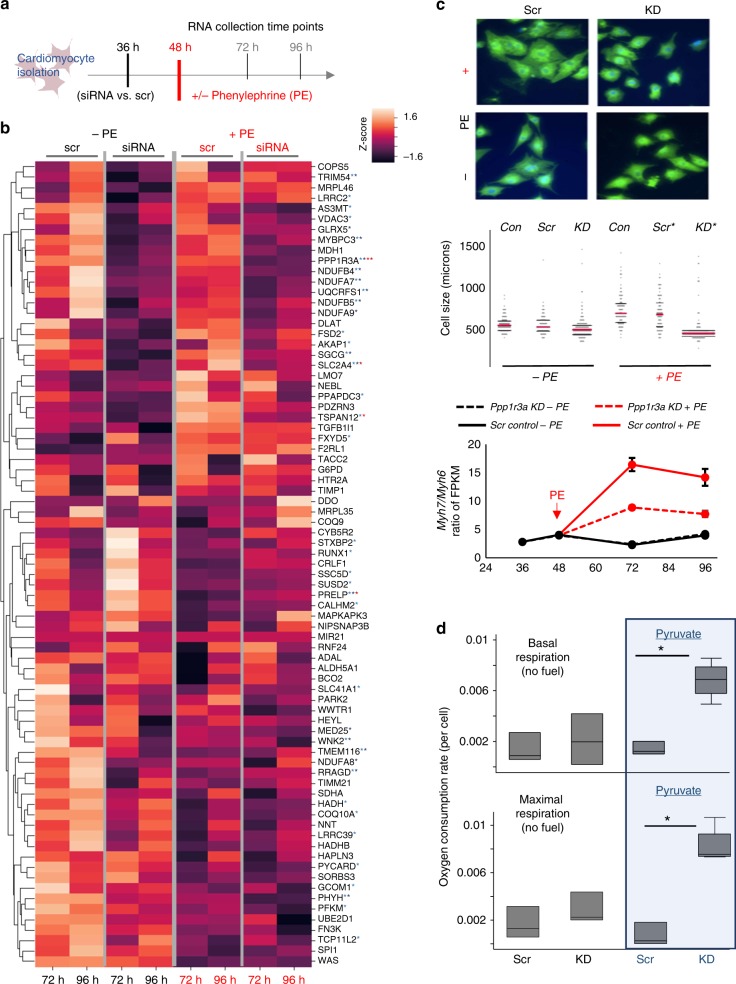
*PPP1R3A* knockdown in vitro reveals metabolic regulation in cardiomyocytes. **a** Experimental design. NRVMs were isolated, transfected with siRNA 36 h later. Phenylephrine or vehicle treatment started at 48 h. RNA was collected at 36, 48, 72 and 96 h after isolation. **b** Clustered heat map of NRVM transcriptional expression of central coordinators in response to *PPP1R3A* knockdown (measured by RNAseq). Expression is shown from NRVMs at 72 and 96 h after isolation normalized to pretreatment expression, and displayed as per-gene *z*-scores. Data from cells with and without phenylephrine (PE) are shown on the left and right sides of the heat map with and without siRNA knockdown as indicated. Stars indicate central coordinators significantly differentially regulated by *PPP1R3A* knockdown (FDR < 0.05, red stars indicate significance in the PE-treated group (red at 72 h, dark red at 96 h) and blue stars indicate significance in the untreated group after *PPP1R3A* knockdown at 72 (light blue) and 96 (dark blue) hours). **c** *PPP1R3A* knockdown protects against hypertrophic stimulus of phenylephrine treatment. Upper panel*:* Cell size measurements of a sample of cells under phenylephrine and normal conditions, with and without *PPP1R3A* KD reveal reduced hypertrophy in NRVMs treated with PE and *PPP1R3A* KD compared to PE-treated cells with and without scramble siRNA transfection (*p* < 1e10^−4^ (ANOVA), * = *p* < 1e10^−3^ by Bonferroni post test. *n* = 100 cells for each group, red bars indicate mean, black bars indicate one standard deviation). Lower panel*: Myh7/Myh6* ratio, a marker for HF, is decreased in *PPP1R3A* KD NRVMs treated with PE compared to those transfected with scrambled siRNA control at 72 and 96 h after isolation (*p* < 1e10^−2^ for both comparisons, error bars represent 95% confidence intervals). **d** Respiratory pyruvate metabolism increases after *PPP1R3A* knockdown. Knockdown of *PPP1R3A* leads to increased basal and maximal respiratory metabolism of pyruvate as measured by oxygen consumption in NRVM (basal respiration: *p* = 0.02, maximal respiration: *p* = 0.005, center line indicates median, box indicates IQR, and whiskers indicate next adjacent value. *n* = 3 biologically independent samples for the siRNA/pyruvate group and *n* = 4 for all other groups). Source data for this figure are provided in a source data file

To investigate the role of *PPP1R3A* in HF, we first determined the effect of perturbing *PPP1R3A* expression via RNA silencing on global gene expression in vitro. We used RNA sequencing to measure global gene expression at various time points with and without *PPP1R3A* knockdown in phenylephrine-treated NRVMs (an in vitro model of cardiomyocyte hypertrophy and HF signaling, Fig. 4a, Supplementary Fig. 6). Global expression of central coordinators significantly changed after knockdown and/or phenylephrine treatment at both time points (* indicates FDR < 0.05, Fig. 4b). The expression of central coordinators (Fig. 3a, b) was highly altered by knockdown of *PPP1R3A* in NRVM both with and without a hypertrophic stimulus (phenylephrine, Fig. 4b). Genes whose change in expression met statistical significance in the global RNA-sequencing dataset were over-represented among these highly dynamic central coordinators (* indicates FDR 0.05, Fig. 4b), providing evidence that PPP1R3A is a central regulator of this network, as manipulation of its expression directly causes significant network perturbation. Consistent with these findings, knockdown of *PPP1R3A* also protected NRVMs against cellular hypertrophy caused by phenylephrine both by measures of cell size and the ratio of *Myh7/Myh6* gene expression over time (Fig. 4c). Taken together, these results indicate that reduction of *PPP1R3A* expression slows cellular HF pathology and its associated signaling in vitro by acting as a central regulator in the network of hypertrophy- and HF-relevant pathways.

Noting that many of the central coordinators significantly regulated by *PPP1R3A* knockdown were metabolic genes (e.g. *GLUT4*, *PFK*, multiple subunits of NADH ubiquinone oxidoreductase and *COQ10*, Fig. 4b) and that prior work has implicated *PPP1R3A* in striated muscle glycogen metabolism, we hypothesized that *PPP1R3A*’s role in the development of HF was related to the metabolic switch from respiratory to glycolytic glucose metabolism observed in failing myocardium. We found that under phenylephrine-treated conditions, *PPP1R3A* knockdown resulted in the alteration of genes critical to glucose metabolism such as glucose transporters *GLUT1* and *GLUT4* (upregulation and downregulation, respectively, Supplementary Fig. 6). Further, under normal NRVM culture conditions, knockdown of *PPP1R3A* induced a significant downregulation of critical regulators of oxidative metabolism, such as the pyruvate dehydrogenases *PDK2*, the carnitine palmitoyltransferase *CPT1B* (Supplementary Fig. 7). Pyruvate dehydrogenase kinases (e.g. *PDK2*) are major molecular drivers of decreased respiratory glucose metabolism in HF via inactivation of pyruvate dehydrogenase^42^. This results in shunting of pyruvate away from the oxidative TCA cycle into lactate, thus decreasing energetic efficiency of glucose metabolism in cardiomyocytes. We therefore hypothesized that decreasing *PPP1R3A* expression would lead to liberation of respiratory metabolism, and found that siRNA-mediated knockdown of *PPP1R3A* leads to increased basal and maximal respiratory metabolism of pyruvate by NRVM as measured by oxygen consumption (*p* = 0.02 (basal respiration) and *p* < 0.01 (maximal respiration) Fig. 4d, blue boxes, see Methods).

### Ppp1r3a^−/−^ mice are protected against LV dysfunction

**Fig. 5:**
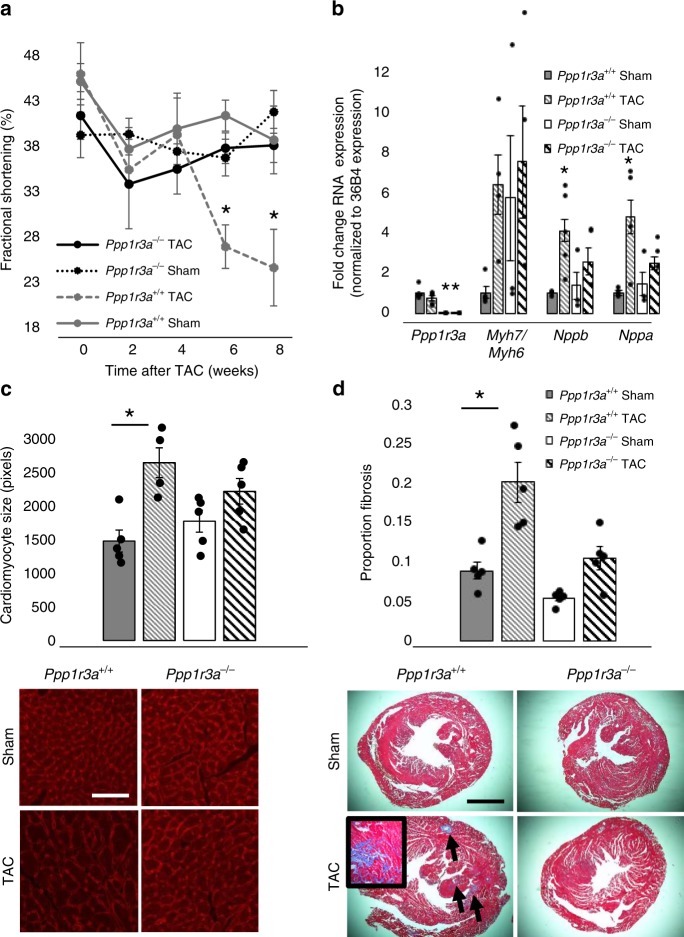
Cardiac effects of *PPP1R3A* ablation in vivo. **a** Fractional shortening is preserved in *Ppp1r3a*^*−/−*^ mice after TAC. At 6 weeks (*n* = 5 animals for all groups), *Ppp1r3a*^*−/−*^ TAC 37.6 ± 4.3%, *Ppp1r3a*^*−/−*^ Sham 36.5 ± 4.7%, *Ppp1r3a*^+/+^ TAC 26.7 ± 9.5%, *Ppp1r3a*^+/+^ Sham 41.2 ± 8.4%, *p* = 0.03 ANOVA and *p* = 0.03 for *Ppp1r3a*^*−/−*^ TAC vs. Sham by Bonferroni post test. At 8 weeks (*n* = 5 for all groups), *Ppp1r3a*^*−/−*^ TAC 37.9 ± 2.8%, *Ppp1r3a*^*−/−*^ Sham 41.6 ± 6.8%, *Ppp1r3a*^+/+^ TAC 24.4 ± 9.0%, *Ppp1r3a*^+/+^ Sham 38.5 ± 2.8%, *p* = 0.002 (ANOVA) and *p* = 0.01 for *Ppp1r3a*^*−/−*^ TAC vs. Sham (Bonferroni post test). **b** Gene expression of HF markers is not increased in *Ppp1r3a*^*−/−*^ animals after TAC: ***PPP1R3a****:* *p* < 0.0001 (ANOVA), *p* < 0.0001 *Ppp1r3a*^+/+^ vs *Ppp1r3a*^*−/−*^ TAC and *Ppp1r3a*^+/+^ vs. *Ppp1r3a*^*−/−*^ Sham, but *p* = 0.41 *Ppp1r3a*^+/+^ TAC vs. Sham (Bonferroni post test). ***MYH7*/*MYH6****:* *p* = 0.11 (ANOVA), *p* = 0.45 *Ppp1r3a*^+/+^ TAC vs. Sham and *p* = 1.0 *Ppp1r3a*^*−/−*^ TAC vs. Sham (Bonferroni post test). ***Nppa****: Ppp1r3a*^+/+^ Sham 1 ± 0.18 (mean fold change ± SD), *Ppp1r3a*^+/+^ TAC: 4.8 ± 2.6, *Ppp1r3a*^*−/−*^ Sham: 1.5 ± 1.1, *Ppp1r3a*^−*/−*^ TAC: 2.5 ± 1.1. *p* = 0.006 (ANOVA), *p* = 0.006 *Ppp1r3a*^+/+^ TAC vs. Sham and *p* = 1.0 *Ppp1r3a*^*−/−*^ TAC vs. Sham (Bonferroni post test). ***Nppb*:** *Ppp1r3a*^+/+^ Sham 1 ± 0.11, *Ppp1r3a*^+/+^ TAC: 4.1 ± 1.9, *Ppp1r3a*^*−/−*^ Sham: 1.4 ± 1.1, *Ppp1r3a*^*−/−*^ TAC: 2.6 ± 1.5. *p* = 0.008 (ANOVA), *p* = 0.01 *Ppp1r3a*^+/+^ TAC vs. Sham and *p* = 1.0 *Ppp1r3a*^*−/−*^ TAC vs. Sham (Bonferroni post test). **c** Preservation of cardiomyocyte size in *Ppp1r3a*^*−/−*^ animals after TAC: *p* = 0.004 (ANOVA), *p* = 0.004 *Ppp1r3a*^+/+^ TAC vs. Sham and *p* = 0.64 *Ppp1r3a*^*−/*−^ TAC vs. Sham (Bonferroni post test). Scale bar indicates 20 μm length. **d** Fibrosis is not increased in *Ppp1r3a*^*−/−*^ animals after TAC (*p* < 0.0001 (ANOVA), *p* = 0.001 *Ppp1r3a*^+/+^ TAC vs. Sham and *p* = 0.22 *Ppp1r3a*^*−/−*^ TAC vs. Sham (Bonferroni post test). Scale bar indicates 200 μm length. *p ≤ 0.05 (Bonferroni post test), TAC transaortic constriction. Error bars indicate standard error of the mean. Source data for this figure are provided in a source data file

We then investigated the effect of *PPP1R3A* on HF in vivo using a model of pressure overload, transaortic constriction (TAC). Compared to *Ppp1r3a*^+/+^, *Ppp1r3a*^−/−^ mice were protected from TAC-induced left-ventricular (LV) dysfunction as measured by fractional shortening (*p* = 0.002 (ANOVA), Fig. 5a). This effect was associated with unchanged levels of the HF markers *Nppa* and *Nppb* in the LV of *Ppp1r3a*^*−/−*^ TAC vs. sham mice compared to the expected TAC-induced increase observed in *Ppp1r3a*^+/+^mice (Fig. 5b, *Nppa*: *p* = 0.006 (ANOVA), *Nppb*: *p* = 0.008 (ANOVA)). No significant difference was found across groups in the ratio of *Myh7/Myh6* expression (Fig. 5b, *p* = 0.11, ANOVA).

In accordance with these findings, there was no difference between *Ppp1r3a*^*−/−*^ TAC and sham in cardiomyocyte size, while *Ppp1r3a*^+/+^ animals showed expected cellular hypertrophy after TAC (Fig. 5c (*p* = 0.004 (ANOVA)). After TAC, heart weight/body weight ratio varied significantly between groups, though TAC comparisons within genotype did not reach statistical significance by Bonferroni post test (*p* = 0.02, ANOVA, *Ppp1r3a*^+/+^ TAC vs. Sham *p* = 0.07 Bonferroni post test, Supplementary Fig. 8). LV fibrosis was also unchanged in *Ppp1r3a*^−/−^ animals after TAC, as opposed to the expected increase in fibrosis seen in *Ppp1r3a*^+/+^ animals in response to TAC (Fig. 5d, *p* < 0.0001 (ANOVA)).

## Discussion

We have constructed a comprehensive gene regulatory map of human heart failure (HF). This effort has been facilitated by a systematic approach to the collection of control and failing heart tissue from the operating rooms of cardiac transplant centers and the resulting measurements have allowed us to describe several previously unrecognized molecular features of HF. Notably, the network structure of HF differs markedly from that of nonfailing heart tissue. Specifically, we find that the control network has a large number of genes (2614) not associated with modules, whereas in the HF network, only 13 genes remained unassociated, providing evidence for increased connectivity in the HF network. Further, in the HF network, there is significant rewiring of genes to new processes (Fig. 1c), and an array of changes in coexpression relationships of central genes to key processes such as sarcomeric structure, excitation−contraction coupling, metabolism, and cardiac remodeling.

The inferred networks also aided in the discovery of new eQTLs in the nonfailing and failing contexts. Notably, we found a greater number of eQTLs in the failing heart, half of which were not previously reported, but that were still implicated in higher phenotype associations in GWAS. In some cases, the expression of local subnetworks of genes were found to be associated with one locus, such as several members of the *TAS2R* G-protein-coupled receptor family, receptors typically associated with the sensation of taste but recently found to be variously expressed in cardiac tissue^33^. Further, these eQTLs were enriched for regulatory annotations, which were more prevalent in the failing heart cohort. Thus, these newly identified eQTLs are not only important for identifying potential regulatory DNA, but also novel molecular actors in HF that would not have been discovered in healthy tissue alone.

In addition to identified eQTLs, comparison of coexpression structure between disease and control networks highlighted genes whose connectivity changed meaningfully between the two cohorts, regardless of change in mean expression. This reveals central coordinating genes in HF that would otherwise be missed by examination of individual gene expression alone. This phenomenon has been observed across human diseases,^43,44^ and highlights a critical feature of coexpression networks: they capture the global complexity of regulation beyond individual changes in expression to identify genes pivotal in disease. Here, our strategy classified genes both by their local connectivity and their network distance to HF-relevant pathways (global connectivity) to identify *PPP1R3A* as a gene with a putative role in HF. *PPP1R3A* would not have been identified without this network approach, given that its own expression is not altered drastically in disease. Subsequent molecular investigations demonstrated its effect on other central coordinators between the control and HF networks as well as a deleterious effect on contractile function in the setting of pressure overload in vivo. Although this gene has not previously been associated with human cardiac disease, studies in both mouse and human have found that loss-of-function mutations in *PPP1R3A* manipulate metabolic pathways in skeletal muscle, and our own analysis implicated it in pyruvate, and other metabolic pathways (Fig. 3c and Fig. S7)^41,45^. Elimination of *PPP1R3A* in a murine model of cardiomyopathy revealed a maladaptive role for this gene in HF, and our in vitro studies highlight the metabolic switch of failing myocardium: toward inefficient glycolytic glucose metabolism and away from the use of pyruvate in respiratory metabolism (Fig. 4d).

While a great strength of this study is its immediate isolation of RNA from freshly explanted human tissue, the resultant networks are not based on gene expression from a single cell type, but rather whole cardiac tissue. While the expression-based networks we use lend themselves to the construction of networks that bridge cell types, we cannot state with certainty that the identified central coordinators are resultant of gene−gene interactions within cardiomyocytes alone, though many of them changed significantly with *Ppp1r3a* knockdown in NRVMs (Fig. 4a). In the same vein, as the causes of HF leading to transplant are diverse, the network-based hypotheses generated by this work are likely to highlight final common pathways of HF resulting from diverse etiologies. This can be viewed as a strength of this work as it is applicable across these multiple etiologies; however, additional studies investigating the early stages of specific HF etiologies will add equally to the literature in future. It must also be noted that, due to the nature of the cohort of control hearts available for transplant, control hearts here are not free of disease (e.g. diabetes, Supplementary Table 1). Though there are more male hearts included in the HF group than control hearts, principal component analysis of gene expression does not reveal segregation by sex (Supplementary Fig. 1). Nevertheless, we controlled for this variable in both the network and eQTL analyses.

Since genome-wide expression studies were introduced, there has been interest in quantifying genes that are significantly differentially expressed, e.g. between failing and nonfailing states. What this linear, unitary approach fails to capture are mechanisms influencing higher order phenotypes reflected in rewiring of transcriptional partners that do not affect expression levels of specific genes. Earlier work has already led to the discovery of central genes using coexpression changes^46,47^. Here, we expanded this use of gene coexpression by exploiting not only gene interaction degree, but also integrated topological network differences and known pathway information. In our HF networks, we have shown how differences between these network topology properties in failing and nonfailing hearts can be used to uncover novel mechanisms and highlight new putative therapeutic targets.

## Methods

### Tissue collection and processing

We established a collaborative multi-institution network with a 24/7 notification system and a team of travel-ready surgeons at major transplant centers to systematize the collection of cardiac tissue from failing hearts and unused heart transplant donors at operating rooms and remote locations. We put in place a series of best practices for procurement of explanted cardiac transplant tissue including harvesting explanted cardiac tissue at the time of cardiac surgery from subjects with HF undergoing transplantation and from unused donor hearts. Hearts were perfused with cold cardioplegia solution prior to cardiectomy to arrest contraction and prevent ischemic damage, and explanted cardiac tissue specimens were flash frozen in liquid nitrogen.

All samples were taken from the left-ventricular free wall at the mid ventricular level (Segments 11 or 12) on the 17-segment model. On some of the occasions, when it was necessary to avoid infarct or peri-infarct tissue in these segments, we obtained tissues that may be closer to the base or apex or more anterior or inferior (segments 1, 4, 5, 6, 7, 10, 16). The septum was never collected. Histopathology using H&E and trichrome staining was used to avoid the use of donor hearts with excess fibrosis or hypertrophy. Immunostaining was not performed.

The institutional review boards at all collection sites (including Stanford, the University of Pennsylvania and the Cleveland Clinic) reviewed and approved the protocols used in this study for procurement and use of human tissue and information. All participants gave informed consent before enrollment.

### Expression and genotype datasets and clinical variables

We performed RNA expression measurements and obtained genotype information in genome-wide markers for 313 patients (177 failing hearts, 136 donor, nonfailing (control) hearts) using Affymetrix expression and Affymetrix Human 6.0 respectively. Clinical variables for each individual were recorded during the course of the research and were compiled using REDCap^48^.

### Data pre-processing, covariate correction, and differential expression

Several technical and sample covariates can bias gene expression values inferred from our microarray data, such as array batch effects and individual ethnicity, gender. These covariates can greatly confound downstream analyses, resulting in false positive and negative associations and reducing the power of statistical analyses. We corrected for these biases in three ways: by normalizing to “reference” probes (control probes with a fixed fluorescence value that control for the geometry and preparation of the array), by applying batch normalization using ComBat^49^, and by correcting for observed covariates (gender, age, and collection site) using robust linear regression. The residuals after these corrections were then used as the gene expression values for downstream analyses. Previously to these corrections, the data was log-transformed to better resemble a normal distribution. Differential expression analysis was performed using the Significance Analysis of Microarrays (SAM)^50^. Genes shown in Supplementary Figure 1B had the highest gene expression difference and were deemed significantly up- or downregulated with a false discovery rate of 5%. QQ plots for differential expression are shown in Supplementary Fig. 4.

### Coexpression networks

We ran the following methods on the covariate-corrected data described in the section above using the top 40% most variable genes (*n* = 7960) and on each cohort separately, except in the case of the joint graphical LASSO (see below).

- WGCNA: We ran the WGCNA pipeline including correlation matrix, TOM transformation, and Dynamic Tree Cut module finding as prescribed^19^ on each network separately.
- Pearson correlation: We obtained the empirical Pearson correlation matrix and performed an absolute correlation coefficient cutoff of *abs*(*R*) > 0.6.
- ARACNe: We ran the method with default parameters and 500 bootstrap iterations for calculating significant gene coexpression relationships.
- Z-score: Otherwise known as the CLR method, we ran the method using the Pearson correlation matrix as input. The output is then a statistical scoring of each interaction for genes *A* and *B* considering all interactions of *A* and *B* against each other gene.
- Joint graphical LASSO (JGL): The JGL has two main parameters—a sparsity penalty to tune how many interactions are found and a group penalty that tries to match the network structure of the two cohorts. We performed a grid search on these parameters and chose the one that maximized the Akaike Information Criterion. This resulted in a very low group penalty of 0.01 and a modicum sparsity penalty of 0.1, which was applied to a standardized matrix of expression values for both cohorts.

While in the end we used WGCNA for downstream analyses as discussed in the main text, we have made all of these networks available at 10.5281/zenodo.2600420.

### eQTL discovery

Prior to eQTL discovery, we used PEER to find hidden covariates that could confound signals in our data as well as filtering any genotypes with major allele frequencies less than 5%. To test associations between gene expression in each cohort separately, we used QTLTools^51^ with an additive model accounting for gender, age, sample site, and the PEER factors as covariates. We corrected for eQTL multiple association testing using a 10,000 permutations per locus in a 2 megabase window and a false discovery rate cutoff of 5%. To select the number of PEER factors, we performed the full analysis multiple times from 1 to 15 PEER factors and observed a saturation of new QTLs being discovered when using 10 factors. To intersect our variants with GTEX and the GWAS catalog, we simply matched based on rsid and position. High-resolution colocalization analysis on coronary artery disease GWAS hits was performed using the eCAVIAR pipeline (see details in Methods)^52^. To find independent eQTLs, we performed LD-pruning (LD, pairwise *r*^2^ < 0.5 within a window of 50 kb) and provide a set of pruned variants in Supplementary Data 1 and 2. QQ plots for eQTL *p* values before and after correction for age, gender and site are provided in Supplementary Fig. 9, and were not significantly inflated by batch correction.

### Cardiac GWAS colocalization analysis

We tested whether any of our eQTLs colocalized with the signals from a publicly available GWAS on coronary artery disease^29^. We ran the eCAVIAR^32^ pipeline using the FINEMAP implementation on all loci with at least one SNP with *p* < 1e-5 in the GWAS and at least one SNP with *p* < 1e-5 in either condition of our eQTL study. We found evidence of colocalization at six genes: *MRAS, TCF21, GPR22, LIPA, ZNF664*, and *EIF2B2. EIF2B2, TCF21*, and *ZNF664* colocalized in both failing and healthy hearts. However, *LIPA* colocalized only in healthy hearts, while *GPR22* and *MRAS* colocalized only in failing hearts. These context-specific colocalizations highlight genes that may contribute to heart disease progression specifically in healthy (*LIPA*) or in already-failing hearts (*GPR22*, *MRAS*). *LIPA* codes for the lysosomal enzyme lipase A. *GPR22* has previously been shown to play a protective role against myocardial^53^. The protein *MRAS* is a muscle-expressed homolog of the Ras oncogene family, currently without any well-characterized mechanism in coronary artery disease.

### Quantifying global and local centrality using network and community membership parameters

The local connectivity metric (*LC*) of any gene *G* was calculated as the difference between max-normalized weighted network degree of *G*. The global connectivity metric (*GC*) of any gene *G* was calculated as the number of gene sets that were significantly differentially enriched between gene rankings of failing and control networks obtained by ordering the genes by their absolute correlation coefficient to *G*.

After inferring the gene coexpression networks for both cohorts, we calculated topological properties for each gene in each network in order to get a sense of a gene’s role in the networks and in the context of known pathways and gene sets. To this end, we defined a gene *g*’s differential global connectivity (*GC*) as the number of curated HF-relevant pathways within its neighborhood (defined by genes lying within a set rank of absolute edge weight to gene *g*) that were significantly enriched in *g*’s neighborhood by the following procedure (see Supplementary Fig. 5):

1. For each pathway, we first ranked the neighbors of *g* by their absolute edge weight (i.e. correlation) to *g* in both the HF and control networks. This resulted into two ranked lists of *g*’s neighbors: HF- and control network specific.
2. For each gene *g*, we plotted the number of network neighbors belonging to a curated list of known HF-relevant pathways from KEGG and Reactome (“HF-relevant neighbors”, see Supplementary Data 9). In this analysis, the number of HF-relevant neighbors of gene *g* in each pathway was plotted on the *y*-axis against progressively larger inclusive neighborhoods. This allowed us to create a distribution of global-HF pathway relatedness taking both known HF-relevant neighbors as well as their distance from gene *g* into account. For example, as shown for *MYH7*, *MYBPC3*, and *PPP1R3A* for the KEGG HCM pathway in Fig. 3d, e, there is a steeper rise in the number of HF-relevant neighbors connected to each gene in the HF network than in the control network.
3. For each gene *g* in each pathway, the difference between the HF network and control network curves was evaluated using the Kolmogorov−Smirnov (KS) statistic (analogous to gene set enrichment analysis^54^) with a Benjamini−Hochberg correction (false discovery rate FDR = 0.01).
4. A gene’s *GC* was then defined as the number of HF-relevant pathways found to be significantly enriched in such manner.

To compare per-gene perturbations in local connectivity between HF and control networks, we defined the change in local connectivity metric *LC* for a gene *g* used for this purpose was calculated as follows:

$$LC\left( g \right) = {\mathrm{{deg}}}\_{\mathrm{{norm}}}\left( {g,\;{\mathrm{{HF}}}\_{\mathrm{{net}}}} \right) - {\mathrm{{deg}}}\_{\mathrm{{norm}}}\left( {g,\;{\mathrm{{Control}}}\_{\mathrm{{net}}}} \right),$$

where deg_norm(*g*, net) is the max-normalized weighted degree of gene *g* in network net (sum of weights of edges that include *g* divided by the maximum network weight across all edges).

Both *LC* and *GC* are then Z-score normalized in order to call coordinator status:

- Central Coordinators have Z-score normalized *LC* and *GC* greater than zero
- Local Coordinators have Z-score normalized *LC* greater than zero but *GC* less than zero
- Pathway Coordinators have Z-score normalized *GC* greater than zero but *LC* less than zero
- Non-coordinators have neither Z-score normalized *GC* or *LC* greater than zero

### Isolation, culture, perturbation, and visualization of cardiac myocytes

All use of animals in this study (including the below in vivo experiments) was reviewed and approved by the Stanford University School of Medicine Institutional Animal Care and Use Committee. NRVMs were isolated from pregnant Sprague−Dawley rats (Charles River) using standard collagenase protocols as described previously and cultured in serum-free DMEM media. In order to attenuate the effects of fibroblast contamination and accentuate metabolic changes, no glucose was added to the media and a final concentration of 20 μM of the fibroblast inhibitor Ara-C (Sigma-Aldrich) was incorporated. Furthermore, for robust expression measurements, at least 1 million cells were plated in a 12-well plate, corresponding to at least 70% confluency. For phenylephrine-treated cells, 50μM of phenylephrine was added 48 h after isolation. For the knockdown experiments, cells were transfected either with an siRNA targeted to PPP1R3A (Stealth siRNA, Invitrogen) or a scrambled siRNA using the RNAiMAX system (Invitrogen) according to the manufacturer instructions; transfections were performed 24 h after isolation. RNA extraction was performed using the Qiagen RNeasy kit according to the manufacturer’s instructions and were DNAse-treated using the DNA-free RNA kit from Zymo research. CDNA was synthesized with the high-capacity cDNA reverse transcription kit from ABI and qRT-PCR assays were performed using KAPA SYBR FAST on a ViiA 7 ABI system.

For visualization, cells were fixed directly in the culture plate wells using 4% PFA in room temperature for 10 min, permeabilized with 0.2% Triton-X in room temperature for 10 min, and blocked with DAKO protein block in room temperature for 30 min. Afterwards, fixed cells were incubated in mouse anti-sarcomeric α-actinin antibody (Sigma) in DAKO antibody buffer overnight at 4 °C. Alexa secondary antibodies were added the next day after PBS washes and the cells were incubated for 1 h at room temperature. Cells were then washed with PBS and the nuclei were stained with DAPI (AntiFade DAPI, Invitrogen). Fluorescent cells were then imaged at 40× or 20× magnification using an Olympus BX-51 inverted fluorescent microscope. Cell area was quantified with ImageJ.

### RNA sequencing and analysis pipeline

After RNA extraction, RNA integrity was checked using a 2100 BioAnalyzer (Agilent); all RNA samples had an RIN of 7.0 or higher. Samples were screened for *PPP1R3A* knockdown efficiency and phenylephrine treatment using qRT-PCR prior to library construction. RNAseq libraries were prepared using the TrueSeq Stranded mRNA kit (Illumina), according to the manufacturers’ instruction. Libraries were barcoded, quality-checked using a 2100 BioAnalyzer and run in rapid run flow cells in a HiSeq 2500 (Illumina), producing at least 30 million paired-end reads.

Sequencing reads were aligned to the *Rattus Norvegicus* rn5 UCSC reference genome using the STAR aligner^55^. Quantification and differential expression analysis of RNAseq data was performed using the Cufflinks package^49^: full transcriptome assembly was performed with Cufflinks, quantified with Cuffquant, and analyzed for differential expression using Cuffdiff. All genes deemed to be significantly up- or downregulated in the main text were called as differentially expressed by Cuffdiff.

### Animals, surgery and phenotyping

Ppp1r3a^*−/−*^ mice (C57Bl6 background) were a generous gift from Anna de Paoli Roach^41^. *Ppp1r3a*^+/+^ animals were C57Bl6 background (Jackson). All procedures involving animal use, housing, and surgeries were approved by the Stanford Institutional Animal Care and Use Committee (IACUC). Animal care and interventions were provided in accordance with the Laboratory Animal Welfare Act.

Twenty male mice (10 *Ppp1r3a*^*−/−*^ and 10 *Ppp1r3a*^+/+^) were randomized to transaortic constriction (TAC) or sham surgery (five in each group). Animals underwent TAC as previously described^50^ at 10 weeks of age. Briefly, mice were anesthetized using an isoflurane inhalation chamber, intubated and ventilated. After surgical exposure of the thoracic aorta, a 6.0 silk suture was placed between the innominate and left carotid arteries to induce a constriction of ∼0.4 mm in diameter. In sham group mice, an identical procedure was conducted, without the constriction of the aorta. One week following TAC, gradients across constriction were measured and were not different between genotypes (*Ppp1r3a*^*−/−*^: 32.1 ± 3.9 mmHg, *Ppp1r3a*^+/+^: 35.6 ± 3.6 mmHg, *p* = 0.16).

In vivo left-ventricular systolic function was evaluated by echocardiography in the short axis view as previously described^50^. Measurements occurred at 1 day prior to surgery (baseline), 7 days and 14 days after surgery and then every 14 days prior to euthanasia and tissue collection at 8 weeks after TAC.

Upon euthanasia, heart weight, body weight and tibia length were measured by standard method (Supplementary Fig. 8). Hearts were paraffin fixed, sectioned and mounted on slides. Trichrome staining as well as immunofluorescence stain for cell membrane (Rhodamine Wheat Germ Agglutinin antibody, 1:200 in PBS, Vector laboratories, Burlingame, CA) were performed for fibrosis and cell size measurements, both of which were performed using ImageJ after image capture at 20×.

### Measurement of oxygen consumption rate

Freshly isolated NRVMs were plated in a 96-well plate at 75,000 cells/well and were maintained in kit medium with 0.5% fetal bovine syndrome. Transfection of siRNA to *PPP1R3A* or scrambled oligonucleotide was performed as described above 5 days after isolation. Media was changed to contain 10% fetal bovine syndrome after transfection.

Seahorse technology (XF96, Flux pack, Agilent technologies # 10-2416100) was used to measure oxygen consumption rate (OCR) 48 h after transfection. Cells were either exposed to base media or media including pyruvate immediately before experiment. Basal metabolism was measured first. Maximal respiration was measured 1 min after delivery of *p*-triflouromethoxyphenylhydrazone (FCCP) (uncoupler of oxidative phosphorylation). After OCR measurements were complete, viable cell number was assayed using PrestoBlue Cell Viability Assay (Thermofisher #A13261), and data were analyzed as OCR per cell.

### Reporting summary

Further information on research design is available in the Nature Research Reporting Summary linked to this article.

## Supplementary information

Supplementary Information

Description of Additional Supplementary Files

Supplementary Data 1

Supplementary Data 2

Supplementary Data 3

Supplementary Data 4

Supplementary Data 5

Supplementary Data 6

Supplementary Data 7

Supplementary Data 8

Supplementary Data 9

Supplementary Data 10

Supplementary Data 11

Reporting Summary

Source Data

## Data Availability

Data that support the findings of this study have been made available. Expression measurements for the human heart samples and clinical variables are available in GEO (accession number GSE57338). eQTLs are available at https://zenodo.org/record/1438557#.W67MS5NKh24. Rat expression measurements are available via Amazon Web Services at http://s3.amazonaws.com/ashleylab-rnaseq/timecourse_analysis.tgz. All other data are contained within the article and its supplementary information or are available upon reasonable request to the corresponding author. All other source data underlying Figs. 4 and 5, and Supplementary Figs. 6 and 8 are provided in a Source Data file.
