# Supplementary material for: Pathologic gene network rewiring implicates PPP1R3A as a central regulator in pressure overload heart failure
Source: Nat Commun. 2019 Jun 24;10:2760. doi: 10.1038/s41467-019-10591-5 (PMC6591478; doi:10.1038/s41467-019-10591-5)
Supplement: Supplementary file 1 — Supplementary Information [file 41467_2019_10591_MOESM1_ESM.docx]

**Pathologic gene network rewiring implicates PPP1R3A as a central regulator in pressure-overload heart failure**

*Cordero et al.*

Supplementary Information

## **Supplementary Tables**

|  | **HF** | | | **Control** | | | **OR [CI], p** |
| --- | --- | --- | --- | --- | --- | --- | --- |
|  | **Yes** | **N** | **%** | **Yes** | **N** | **%** |  |
| **Age** | 55.8 +/- 0.8 | 177 |  | 49.3 +/- 1.3 | 136 |  | <0.0001 |
| **Gender (male)** | 145 | 177 | 82 | 73 | 136 | 54 | 3.9 [2.3,6.7],<0.0001 |
| **Weight (kg)** | 82.7+/- 15.4 | 175 |  | 79.0+/-25.9 | 134 |  | 0.11 |
| **Diabetes** | 47 | 177 | 27 | 17 | 134 | 13 | 2.5,[1.3,4.9], 0.003 |
| **IDDM** | 13 | 47 | 28 | 3 | 17 | 18 | 2.7 [0.7,15.5], 0.12 |
| **HTN** | 83 | 174 | 48 | 64 | 134 | 48 | 1.0 [0.61 ,1.6] 0.99 |
| **SCD** | 7 | 62 | 11 |  |  |  |  |
| **ICD shock** | 27 | 69 | 39 |  |  |  |  |
| **AF** | 53 | 176 | 30 | 5 | 58 | 9 |  |
| **VT/VF** | 83 | 177 | 47 | 1 | 55 | 2 |  |
| **Sustained VT** | 32 | 69 | 46 |  |  |  |  |
| **ACE-Inhibitor** | 126 | 177 | 71 | 18 | 67 | 27 | 6.7, [3.4, 13.4], <0.0001 |
| **Angiotensin Receptor Blocker** | 57 | 177 | 32 | 4 | 56 | 7 | 6.2 [2.1, 24.5], 0.0002 |
| **Beta-Blocker** | 92 | 177 | 52 | 18 | 61 | 30 | 2.6, [1.3,5.1], 0.002 |
| **Aldosterone Antagonist** | 34 | 69 | 49 |  |  |  |  |
| **Milrinone** | 86 | 169 | 51 |  |  |  |  |
| **Dopamine** | 1 | 69 | 1 |  |  |  |  |
| **Dobutamine** | 56 | 159 | 35 |  |  |  |  |
| **Digoxin** | 113 | 177 | 64 |  |  |  |  |
| **Diuretics** | 126 | 177 | 71 |  |  |  |  |
| **Hydralazine** | 22 | 177 | 12 |  |  |  |  |
| **Nitrates** | 78 | 177 | 44 |  |  |  |  |
| **CABG** | 64 | 177 | 36 |  |  |  |  |

**Supplementary Table 1. Clinical characteristics of MAGNet Patients included in study.** N represents number of subjects for whom data was available (of 177 HF and 136 Control). IDDM: History of insulin use. HTN: History of hypertension. SCD: History of sudden cardiac death/arrest, ICD: Implantable cardiac defibrillator. AF: history of atrial fibrillation. VT/VF: History of documented ventricular tachycardia or ventricular fibrillation. CABG: history of coronary artery bypass grafting. Medication classes indicate any history of patient taking these medications by chart review.


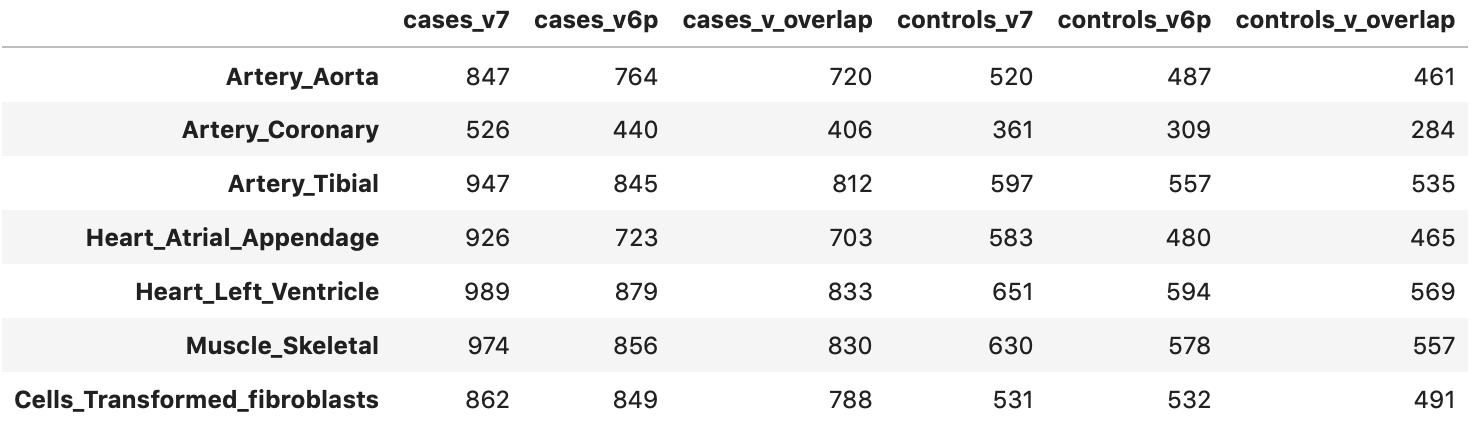


**Supplementary Table 2. Comparison between eQTL overlap between GTEx versions.** While the analysis was conducted on GTEx v6p, we also compared the overlap against v7. Unlike previous releases of GTEx that genotyped using Illumina's OMNI 5M + imputation to 1000 Genomes Project Phase, v7 used whole genome sequencing, which should yield more accurate results. We overlapped the eQTLs that we derived with eQTLs from various heart-related tissues in GTEx. The number of eQTLs that overlapped increased by about 102 for the cases (HF) and 44 for controls between v6p and v7.

## **Supplementary Figures**


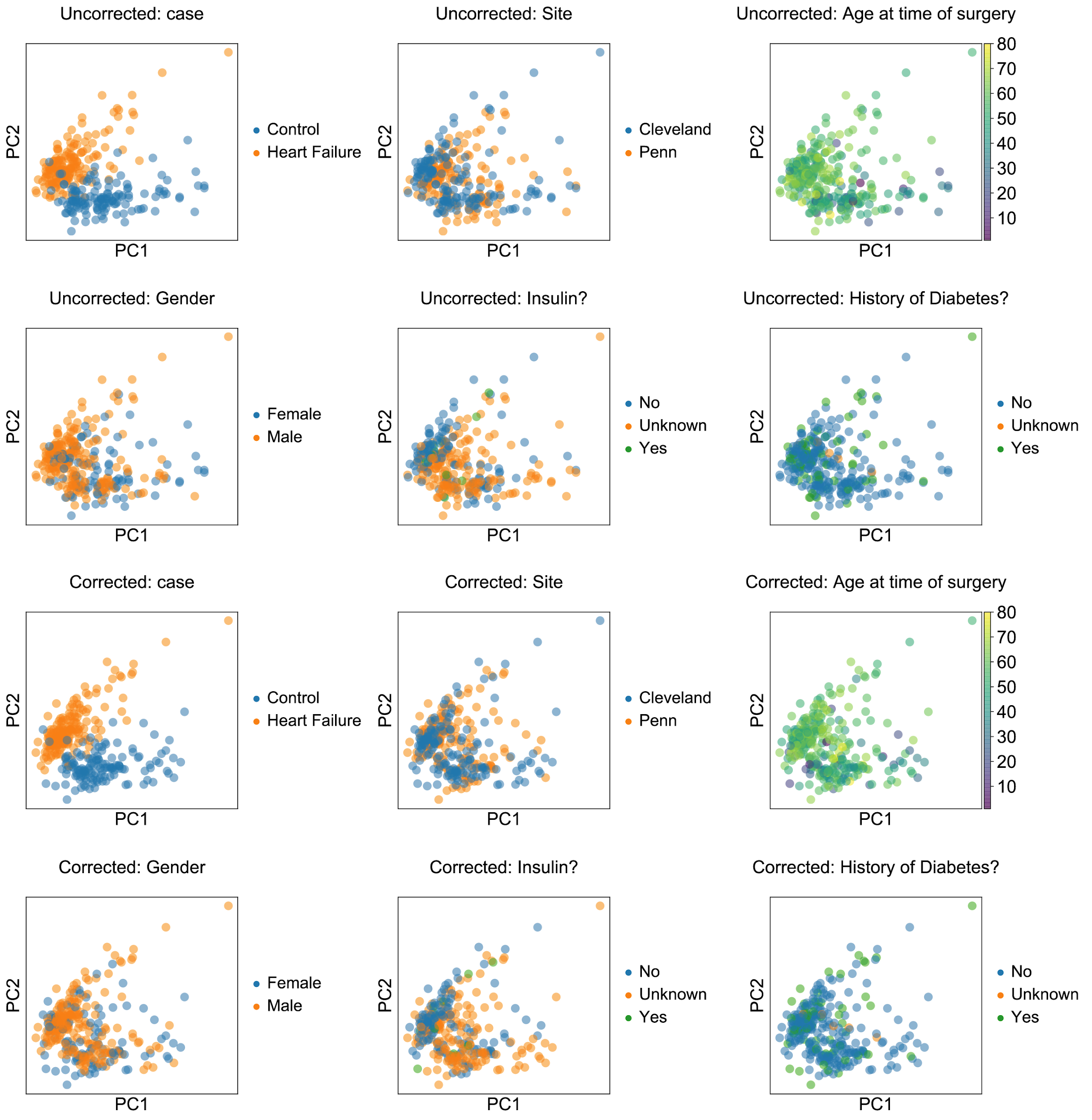


**Supplementary Figure 1:** Principal Component Analysis showing lack of segregation of potential clinical confounders with components of gene expression. Patients are labeled using covariates and comorbidities plotted before and after batch correction on the first two principal components.


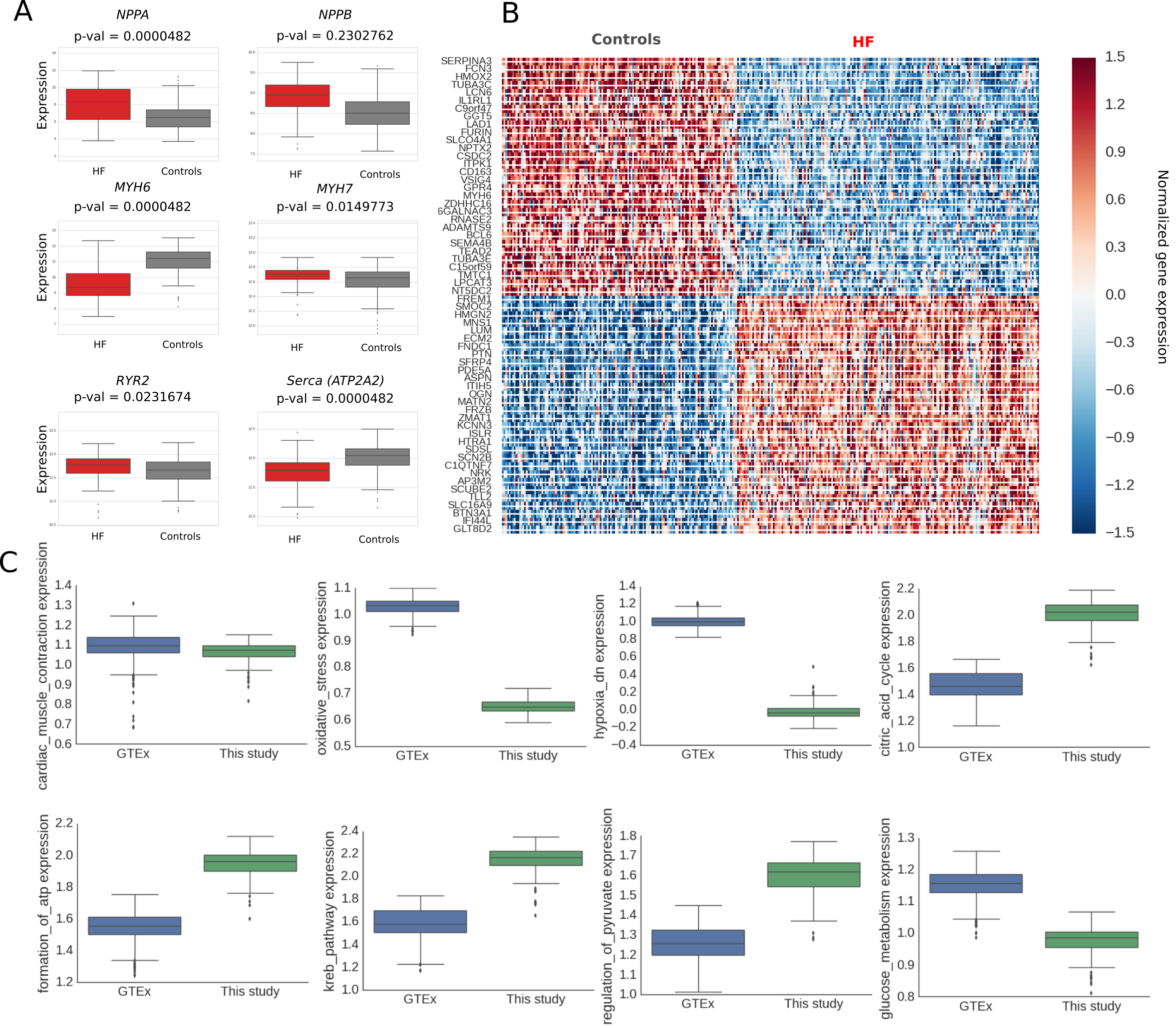


**Supplementary Figure 2: (A)** Expression of various genes involved in heart failure in the failing and non-failing control cohorts. (P-values as indicated). **(B)** Top differentially expressed genes between failing and non-failing controls. **(C)** Mean expression of various gene sets, including metabolic pathways and oxidative stress/hypoxia genes of the GTEx post-mortem samples (blue) and the control cohort in this study (green) obtained from heart transplant donor hearts. Error bars indicate one standard deviation.


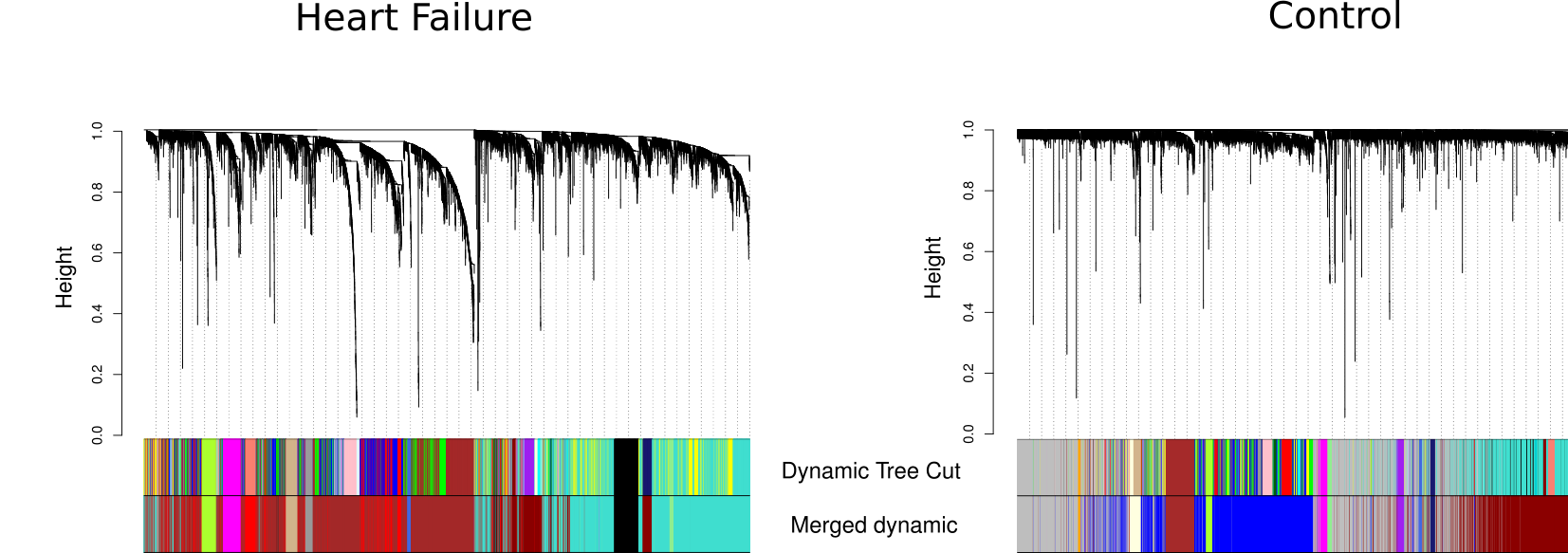


**Supplementary Figure 3: Dendrograms from WGCNA HF (left) and control (right) networks.** While the number of modules found in each network are similar (12 for HF and 13 for controls), dendrograms visually indicate a more cohesive structure in HF compared to controls, with 2614 genes unassigned to any module in the control (gray module) compared to only 13 in HF.

**A**


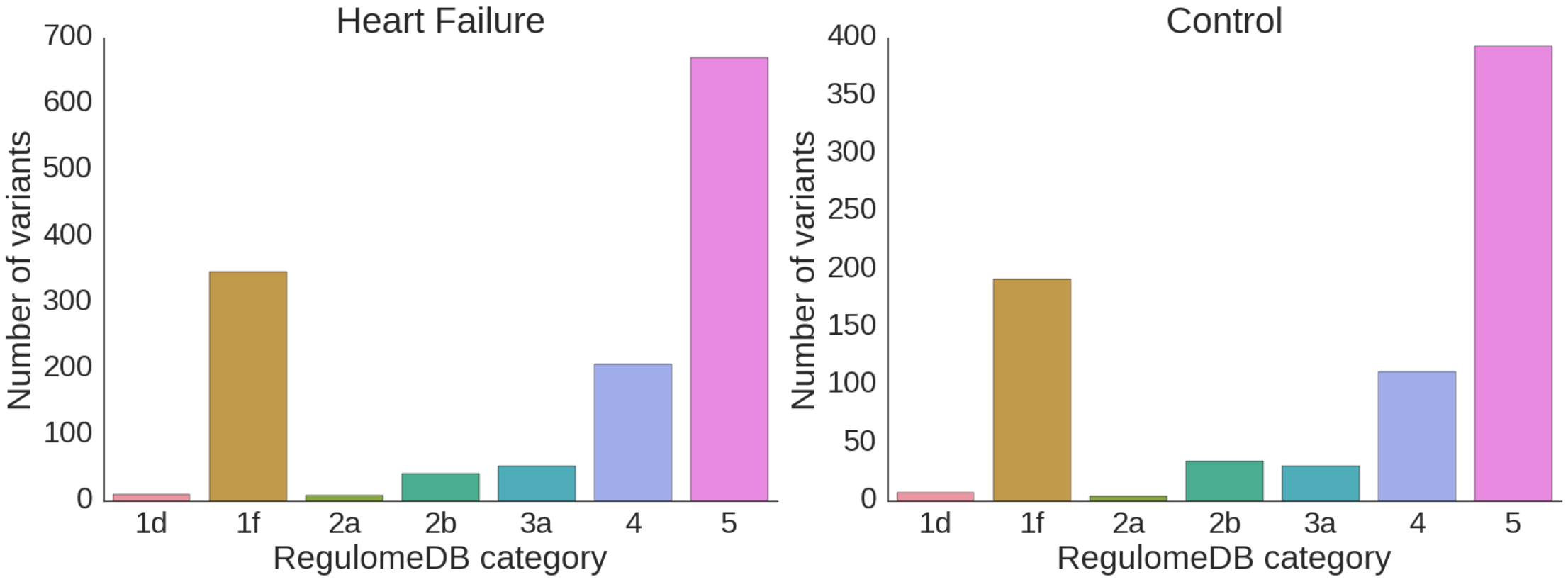


**B**

**
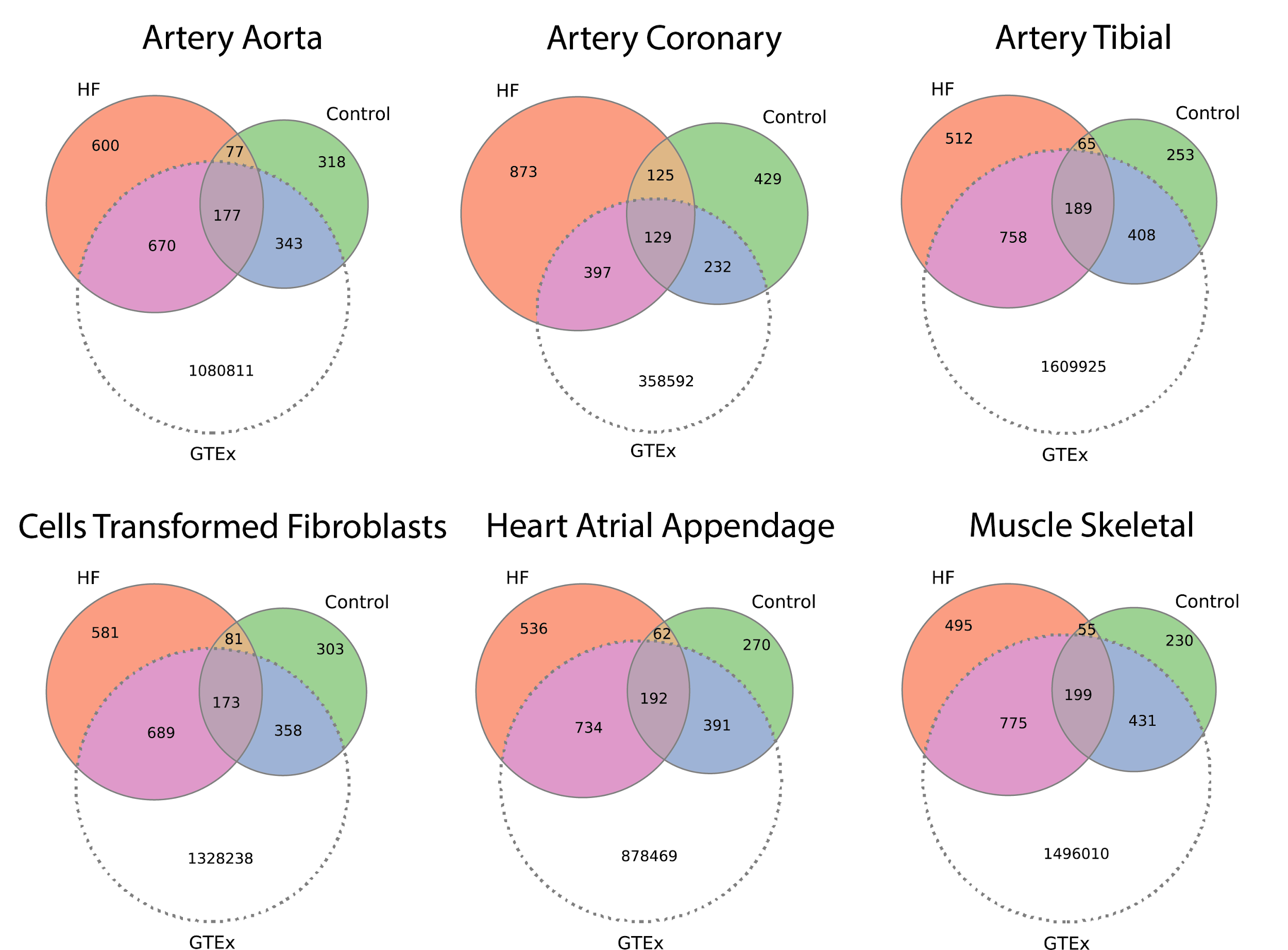
**

**Supplementary Figure 4:** **(A)** Number of eQTLs for each network for which there were RegulomeDB annotations within a 50 base pair window. RegulomeDB categories are defined as follows: category 1 are known eQTLs with ENCODE DNase sensitivity peaks (1f) and TF binding data (1d); category 2 only have evidence of TF binding (2b) and DNase sensitivity peaks as well as a matching TF binding motif (2a); category 3a have predicted TF binding and a TF motif as well as DNase peak; category 4 have predicted TF binding and a DNase peak; and category 5 have either predicted TF binding or a DNase peak. **(B)** Venn diagrams illustrating overlap of HF and Control network eQTLs by relevant tissue type in GTEX.


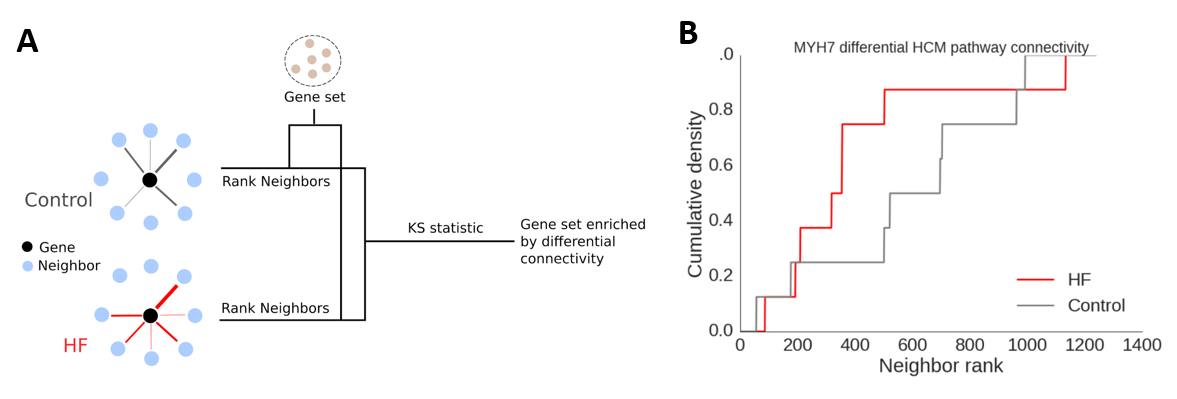


**Supplementary Figure 5:** Calculation of the differential global connectivity (*GC*) of a gene between the HF and Control networks. **(A)** All neighbors of each gene are ranked by edge weight (i.e. network distance). These lists are cross-referenced with the set of genes in manually curated HF-relevant pathways from KEGG and Reactome (Here referred to as “Gene set,” Supplemental File S***). **(B)** The number of these globally HF-relevant neighbors within a given edge-weight threshold is then plotted against expanding edge weight thresholds for the HF (red) and control (grey) networks. A KS statistic is then applied. The *GC* for each gene is the normalized number of pathways to which it is significantly differentially connected (BH corrected FDR < 0.01) based on this test.
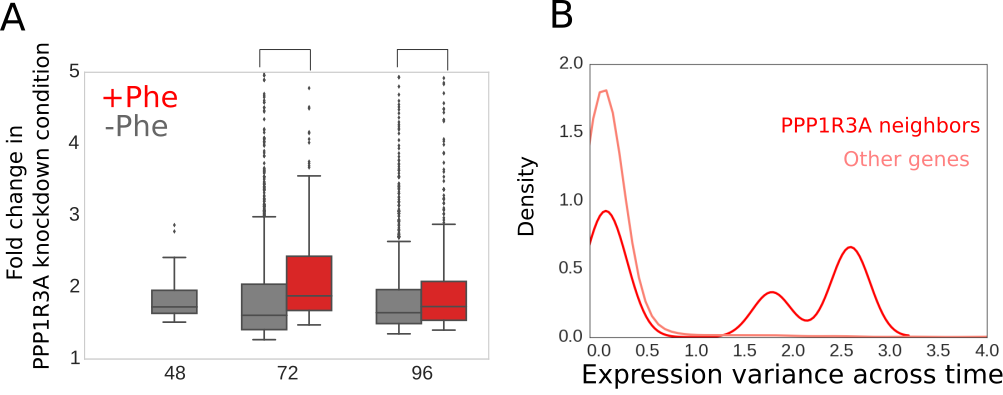

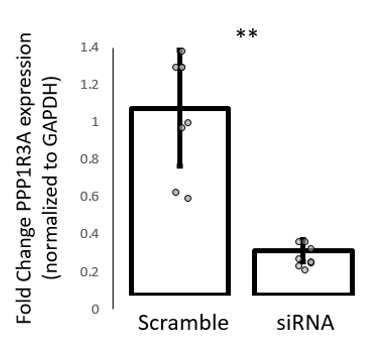


Hours after isolation

**Supplementary Figure 6: (Left)** qRT-PCR validation of PPP1R3A knockdown by siRNA (data shown is 72 hours after isolation, p = 7.8E-06 by student’s T-test, error bars indicate SEM). **(Right)** Fold change distributions within timepoints between Phenylephrine induced NRVMs and controls under *PPP1R3A* knockdown. Center line indicates mean, box indicates IQR and whiskers indicate 95% CI. Source data are provided as a source data file.


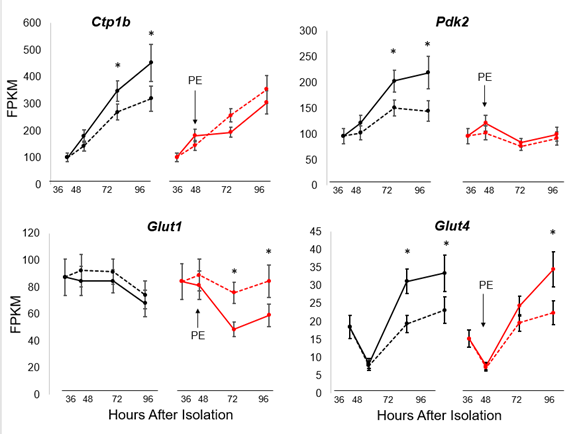


**Supplementary Figure 7:**  Differences in expression levels of key metabolic genes in NRVM after siRNA-mediated *PPP1R3A* knockdown (dashed lines) with and without phenylephrine treatment (red lines). Error bars indicate SD.


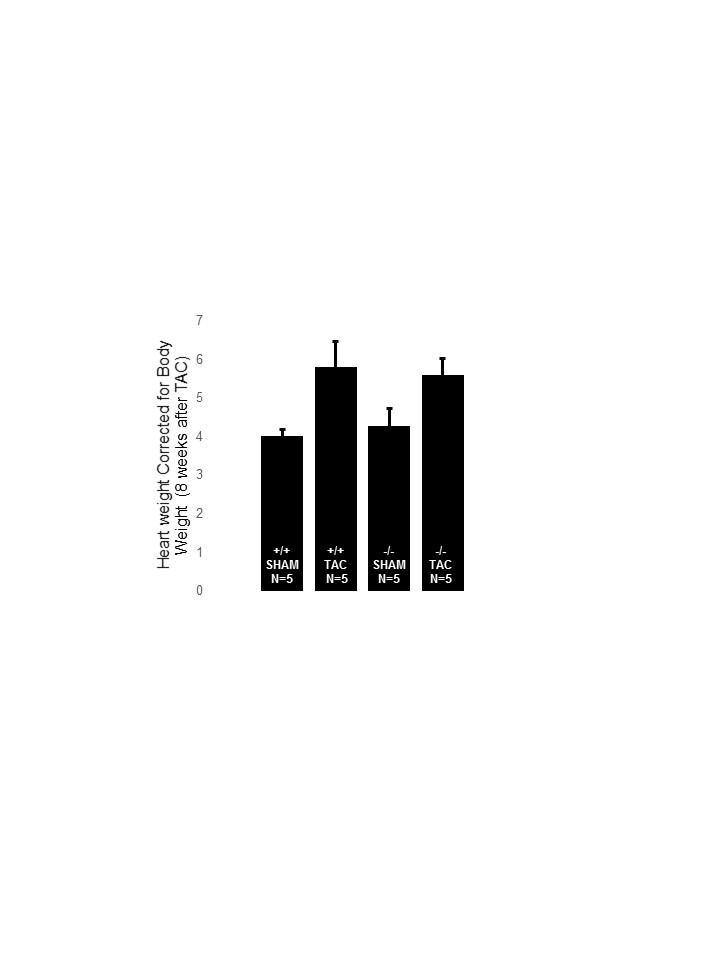


**Supplementary Figure 8:** Heart weight differences (normalized to body weight) between wild type and *Ppp1r3a^-/-^* mice after TAC; p=0.02 (ANOVA). *Ppp1r3a^+/+^* TAC vs Sham p=0.07 (Bonferroni post test). *Ppp1r3a^-/-^* TAC vs Sham p=0.35 (Bonferroni post test). Error bars indicate SEM. Source data are provided as a source data file.

**
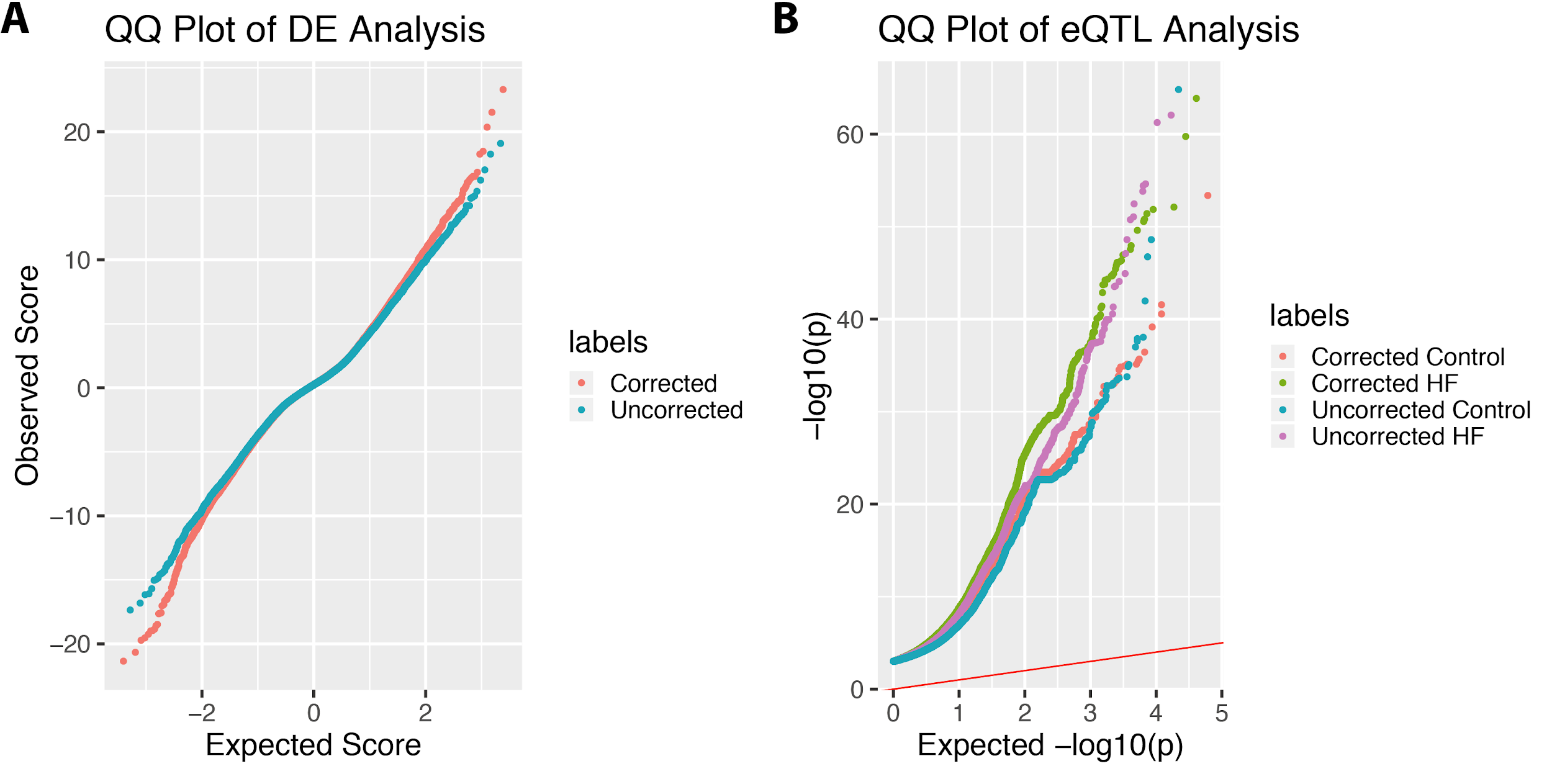
**

**Supplementary Figure 9: (A)** QQ plots of the differential expression analysis before and after batch correction. Batch correction does not appear to significantly skew p-values. **(B)** QQ plots of the eQTL analysis before and after batch correction. Batch correction does not appear to significantly skew p-values.
