# Supplementary material for: Pathologic gene network rewiring implicates PPP1R3A as a central regulator in pressure overload heart failure
Source: Nat Commun. 2019 Jun 24;10:2760. doi: 10.1038/s41467-019-10591-5 (PMC6591478; doi:10.1038/s41467-019-10591-5)
Supplement: Supplementary file 2 — Description of Additional Supplementary Files [file 41467_2019_10591_MOESM2_ESM.docx]

**Description of Additional Supplementary Files**

**File Name: Supplementary Data 1 & 2**

**Description:** To find independent eQTLs, we performed LD-pruning (LD, pairwise r 2 < 0.5 within a window of 50 kb) and provide a set of pruned variants for cases and controls respectively.

**File Name: Supplementary Data 3 & 4**

**Description:** Full differential expression analysis for failing and non-failing human cardiac tissue (Upregulated genes in 3 and downregulated genes in 4).

**File Name: Supplementary Data 5 & 6**

**Description:** Full WGCNA derived gene module descriptions and Benjamini-Hochberg adjusted enrichment p-values for cases and controls respectively.

**File Name: Supplementary Data 7 & 8**

**Description:** Overlap between HF eQTLs and GWAS catalogue for cases and controls respectively.

**File Name: Supplementary Data 9**

**Description:** Curated list of known HF-relevant pathways from KEGG and Reactome for defining global connectivity.

**File Name: Supplementary Data 10**

**Description:** Genes with Associated Coordinator Status (non-, local, pathway or centralcoordinator) Supplementary Data.

**File Name: Supplementary Data 11**

**Description:** Manually curated genes associated with heart failure relevant processes for

Figure 1.
