# Supplementary material for: Pathologic gene network rewiring implicates PPP1R3A as a central regulator in pressure overload heart failure
Source: Nat Commun. 2019 Jun 24;10:2760. doi: 10.1038/s41467-019-10591-5 (PMC6591478; doi:10.1038/s41467-019-10591-5)
Supplement: Supplementary file 14 — Reporting Summary [file 41467_2019_10591_MOESM14_ESM.pdf]

nature research

Corresponding author(s): Dr. Loh Yuin-Han

Last updated by author(s): May 17, 2019

# Reporting Summary

Nature Research wishes to improve the reproducibility of the work that we publish. This form provides structure for consistency and transparency in reporting. For further information on Nature Research policies, see Authors & Referees and the Editorial Policy Checklist.

## Statistics

For all statistical analyses, confirm that the following items are present in the figure legend, table legend, main text, or Methods section.

| n/a | Confirmed | |
|---|---|---|
| ☐ | ☒ | The exact sample size (*n*) for each experimental group/condition, given as a discrete number and unit of measurement |
| ☐ | ☒ | A statement on whether measurements were taken from distinct samples or whether the same sample was measured repeatedly |
| ☐ | ☒ | The statistical test(s) used AND whether they are one- or two-sided<br>*Only common tests should be described solely by name; describe more complex techniques in the Methods section.* |
| ☒ | ☐ | A description of all covariates tested |
| ☒ | ☐ | A description of any assumptions or corrections, such as tests of normality and adjustment for multiple comparisons |
| ☐ | ☒ | A full description of the statistical parameters including central tendency (e.g. means) or other basic estimates (e.g. regression coefficient) AND variation (e.g. standard deviation) or associated estimates of uncertainty (e.g. confidence intervals) |
| ☐ | ☒ | For null hypothesis testing, the test statistic (e.g. *F*, *t*, *r*) with confidence intervals, effect sizes, degrees of freedom and *P* value noted<br>*Give P values as exact values whenever suitable.* |
| ☒ | ☐ | For Bayesian analysis, information on the choice of priors and Markov chain Monte Carlo settings |
| ☒ | ☐ | For hierarchical and complex designs, identification of the appropriate level for tests and full reporting of outcomes |
| ☒ | ☐ | Estimates of effect sizes (e.g. Cohen's *d*, Pearson's *r*), indicating how they were calculated |

*Our web collection on statistics for biologists contains articles on many of the points above.*

## Software and code

Policy information about availability of computer code

| | |
|---|---|
| Data collection | MXexpress |
| Data analysis | HOMER, r3Cseq, MACS2, STAR, SeqMonk, cTen |

For manuscripts utilizing custom algorithms or software that are central to the research but not yet described in published literature, software must be made available to editors/reviewers. We strongly encourage code deposition in a community repository (e.g. GitHub). See the Nature Research guidelines for submitting code & software for further information.

## Data

Policy information about availability of data

All manuscripts must include a data availability statement. This statement should provide the following information, where applicable:

- Accession codes, unique identifiers, or web links for publicly available datasets
- A list of figures that have associated raw data
- A description of any restrictions on data availability

Sequencing data that support the findings of this study have been deposited in GEO with the accession code GSE119951.

# Field-specific reporting

Please select the one below that is the best fit for your research. If you are not sure, read the appropriate sections before making your selection.

☒ Life sciences ☐ Behavioural & social sciences ☐ Ecological, evolutionary & environmental sciences

For a reference copy of the document with all sections, see nature.com/documents/nr-reporting-summary-flat.pdf

# Life sciences study design

All studies must disclose on these points even when the disclosure is negative.

| | |
|---|---|
| Sample size | Our study didn't involve human subjects or animals. We used cell lines and prepared the NGS libraries based on their standard procedure. |
| Data exclusions | No data is excluded in this paper |
| Replication | All attempts of replication were successful |
| Randomization | This is not applicable to our study because we were working on cell lines |
| Blinding | This is not applicable to our study because we were working on cell lines |

# Reporting for specific materials, systems and methods

We require information from authors about some types of materials, experimental systems and methods used in many studies. Here, indicate whether each material, system or method listed is relevant to your study. If you are not sure if a list item applies to your research, read the appropriate section before selecting a response.

## Materials & experimental systems

| n/a | Involved in the study | |
|---|---|---|
| ☐ | ☒ | Antibodies |
| ☐ | ☒ | Eukaryotic cell lines |
| ☒ | ☐ | Palaeontology |
| ☒ | ☐ | Animals and other organisms |
| ☒ | ☐ | Human research participants |
| ☒ | ☐ | Clinical data |

## Methods

| n/a | Involved in the study | |
|---|---|---|
| ☐ | ☒ | ChIP-seq |
| ☒ | ☐ | Flow cytometry |
| ☒ | ☐ | MRI-based neuroimaging |

## Antibodies

| | |
|---|---|
| Antibodies used | Anti-Oct4(ab19857, abcam), Anti-STAT3 (phospho Y705) antibody(ab76315, abcam), Anti-STAT3 antibody (ab31370, abcam),anti-FLAG(F1804, sigma) |
| Validation | Anti-Oct4(ab19857, abcam) is validated for IF, Anti-STAT3 (phospho Y705) antibody(ab76315, abcam) is validated for ChIP, Anti-STAT3 antibody (ab31370, abcam) is validated for ChIP,anti-FLAG(F1804, sigma) is validated for IF and IP |

## Eukaryotic cell lines

Policy information about cell lines

| | |
|---|---|
| Cell line source(s) | ES-E14TG2a (ATCC® CRL-1821™), NIH/3T3 (ATCC® CRL-1658™) and MEF (C57BL/6) [MEF-BL/6-1] (ATCC® SCRC-1008™) |
| Authentication | None of the cell lines used have been authenticated. |
| Mycoplasma contamination | All cell lines tested negative for mycoplasma contamination |
| Commonly misidentified lines (See ICLAC register) | No commonly misidentified cell lines were used. |

## ChIP-seq

### Data deposition

☒ Confirm that both raw and final processed data have been deposited in a public database such as GEO.

☒ Confirm that you have deposited or provided access to graph files (e.g. BED files) for the called peaks.

| | |
|---|---|
| Data access links *May remain private before publication.* | https://www.ncbi.nlm.nih.gov/geo/query/acc.cgi?acc=GSE119951 Reviewer Token: sferauwonhsbjul |
| Files in database submission | Stat3_shEV_R1.fastq.gz |

| | |
|---|---|
| Files in database submission | Stat3_shEV_R2.fastq.gz<br>Stat3_shStat3_R1.fastq.gz<br>Stat3_shStat3_R2.fastq.gz<br>Stat3_shLrrc31-1_R1.fastq.gz<br>Stat3_shLrrc31-1_R2.fastq.gz<br>Stat3_shLrrc31-2_R1.fastq.gz<br>Stat3_shLrrc31-2_R2.fastq.gz<br>Stat3_shEV_Input_R1.fastq.gz<br>Stat3_shEV_Input_R2.fastq.gz<br>Stat3_shStat3_Input_R1.fastq.gz<br>Stat3_shStat3_Input_R2.fastq.gz<br>Stat3_shLrrc31-1_Input_R1.fastq.gz<br>Stat3_shLrrc31-1_Input_R2.fastq.gz<br>Stat3_shLrrc31-2_Input_R1.fastq.gz<br>Stat3_shLrrc31-2_Input_R2.fastq.gz<br>Stat3_shEV.txt<br>Stat3_shStat3.txt<br>Stat3_shLrrc31-1.txt<br>Stat3_shLrrc31-2.txt |
| Genome browser session (e.g. UCSC) | http://genome.ucsc.edu/s/chadi/Stat3_ChIP%2DSeq_E14 |

## Methodology

| | |
|---|---|
| Replicates | Stat3_shLrrc31-1 and Stat3_shLrrc31-2 are biogical replicates. The jaccard index between these two samples is > 0.1 |
| Sequencing depth | Stat3_shEV; Total Reads: 40544796; Unique Mapped Reads: 27912537; Length: 101; Paired-end<br>Stat3_shStat3; Total Reads: 23598170; Unique Mapped Reads: 16127610; Length: 101; Paired-end<br>Stat3_shLrrc31-1; Total Reads: 48861398; Unique Mapped Reads: 34131942; Length: 101; Paired-end<br>Stat3_shLrrc31-2; Total Reads: 31773626; Unique Mapped Reads: 20676612; Length: 101; Paired-end<br>Stat3_shEV_Input; Total Reads: 43412077; Unique Mapped Reads: 30205065; Length: 101; Paired-end<br>Stat3_shStat3_Input; Total Reads: 41006216; Unique Mapped Reads: 28325468; Length: 101; Paired-end<br>Stat3_shLrrc31-1_Input; Total Reads: 30579659; Unique Mapped Reads: 21079308; Length: 101; Paired-end<br>Stat3_shLrrc31-2_Input; Total Reads: 28070823; Unique Mapped Reads: 18619280; Length: 101; Paired-end |
| Antibodies | Anti-STAT3 antibody (ab31370, abcam) |
| Peak calling parameters | ##Mapping Fastq Files:<br>/Path/To/STAR --runThreadN 10 --genomeDir /Path/To/STAR/Genome/Index/mm9/ --readFilesIn /Path/To/Sample_R1.fastq.gz /Path/To/Sample_R2.fastq.gz --readFilesCommand zcat --outFileNamePrefix ./Path/To/Output --outSAMtype BAM SortedByCoordinate --outFilterMultimapNmax 2 --outFilterMismatchNmax 3 --alignIntronMax 1 --alignEndsType EndToEnd<br><br>###Creation of Tag Directories:<br>makeTagDirectory /Path/To/Tag/Directory/ /Path/To/Bam/File<br><br>### Peak Calling<br>findPeaks /Path/To/Tag/Directory -style factor -o auto -i /Path/To/Input/Tag/Directory |
| Data quality | To be considered for downstream analyses, a read must be: (1) uniquely mapped to Genome. (2) Contains less than 3 mismatches. (3) have a mean quality score above 30.<br><br>Peaks should pass the following thresholds to be considered for downstream analysis: (1) FDR < 5%. (2) Minimum 4-folds enrichment over inputs. (3) Minimum 4-fold enrichment over local background (sorrounding 10 kb regions)<br><br>Number of Peaks with FDR < 5% and minimum 5 fold enrichment over input:<br>Stat3_shEV: 14984<br>Stat3_shStat3: 8590<br>Stat3_shLrrc31-1: 4397<br>Stat3_shLrrc31-2: 6324 |
| Software | We used STAR v2.5.3a to map the fastq files to mm9 genome assembly. HOMER v4.10 was used to create tag directories and call peaks |
